# Immune response to viscerotropic *Leishmania*: a comprehensive review

**DOI:** 10.3389/fimmu.2024.1402539

**Published:** 2024-09-18

**Authors:** Lorenzo Lodi, Marta Voarino, Silvia Stocco, Silvia Ricci, Chiara Azzari, Luisa Galli, Elena Chiappini

**Affiliations:** ^1^ Department of Health Sciences, University of Florence, Florence, Italy; ^2^ Immunology Unit, Department of Pediatrics, Meyer Children’s Hospital IRCCS, Florence, Italy; ^3^ Infectious Diseases Unit, Department of Pediatrics, Meyer Children’s Hospital IRCCS, Florence, Italy

**Keywords:** immunity, visceral, leishmania, asymptomatic, symptomatic, persistent, HLH, PKDL

## Abstract

*L. donovani* and *L. infantum* infections are associated with a broad clinical spectrum, ranging from asymptomatic cases to visceral leishmaniasis (VL) with high mortality rates. Clinical manifestations such as post-kala-azar dermal leishmaniasis (PKDL) and visceral leishmaniasis-associated hemophagocytic lymphohistiocytosis-mimic (VL-associated HLH-mimic) further contribute to the diversity of clinical manifestations. These clinical variations are intricately influenced by the complex interplay between the host’s immune response and the parasite’s escape mechanisms. This narrative review aims to elucidate the underlying immunological mechanisms associated with each clinical manifestation, drawing from published literature within the last 5 years. Specific attention is directed toward viscerotropic *Leishmania* sinfection in patients with inborn errors of immunity and acquired immunodeficiencies. In VL, parasites exploit various immune evasion mechanisms, including immune checkpoints, leading to a predominantly anti-inflammatory environment that favors parasite survival. Conversely, nearly 70% of individuals are capable of mounting an effective pro-inflammatory immune response, forming granulomas that contain the parasites. Despite this, some patients may experience reactivation of the disease upon immunosuppression, challenging current understandings of parasite eradication. Individuals living with HIV and those with inborn errors of immunity present a more severe course of infection, often with higher relapse rates. Therefore, it is crucial to exclude both primary and acquired immune deficiencies in patients presenting disease relapse and VL-associated HLH-mimic. The distinction between VL and HLH can be challenging due to clinical similarities, suggesting that the nosological entity known as VL-associated HLH may represent a severe presentation of symptomatic VL and it should be considered more accurate referring to this condition as VL-associated HLH-mimic. Consequently, excluding VL in patients presenting with HLH is essential, as appropriate antimicrobial therapy can reverse immune dysregulation. A comprehensive understanding of the immune-host interaction underlying *Leishmania* infection is crucial for formulating effective treatment and preventive strategies to mitigate the disease burden.

## 1 Introduction

Leishmaniasis is a parasitic infection caused by an obligate intracellular protozoan parasite belonging to the genus *Leishmania* of the family *Trypanosomatidae*. It is considered a tropical disease, endemic in tropical and subtropical regions globally, including the Mediterranean basin.

*Leishmania* behaves mainly as a vector-borne zoonotic parasite, transmitted between animal reservoirs (i.e. dogs and rodents) and the human host by the Phlebotomus sandfly. The infected female sandfly introduces parasites, in the form of promastigotes, into the host’s bloodstream during blood meals. These promastigotes are engulfed by macrophages and other mononuclear phagocytic cells where they transform into amastigotes, the actively replicating form of the parasite, ultimately leading to systemic dissemination through the reticuloendothelial system. When an infected host is bitten by another sandfly, the ingested amastigotes transform back into promastigotes, facilitating transmission to another mammalian host during a subsequent blood meal. In addition, *Leishmania* can also be transmitted by blood derivatives and by vertical transmission (1–3).

*Leishmania* infection manifests in three distinct clinical forms: cutaneous leishmaniasis, mucocutaneous leishmaniasis and visceral leishmaniasis (VL), each caused by different *Leishmania* strains. Within this review, we will address the host-parasite immune interaction in VL which is caused by *L. donovani* and *L. infantum* (also known as *L. chagasi*) strains. Such strains are hereafter referred to as viscerotropic strains. According to the World Health Organization (WHO), there are 50,000 to 90,000 new cases occurring annually worldwide, although this data may be underestimated due to the potential underdiagnosis and underreporting in tropical regions (4).

VL is a disease with an extremely broad clinical spectrum ranging from asymptomatic to clinically significant infections with high mortality. The clinical manifestations are marked by insidious onset of persistent fever, coupled with hepatosplenomegaly and pancytopenia. These symptoms emerge after an extended incubation period, typically lasting between 2 to 6 months on average, but occasionally spanning across several years. According to the WHO, symptomatic VL has a mortality rate of over 95% when untreated (4).

The idea that the same parasitic strain can elicit vastly different clinical responses in distinct individuals suggests a remarkably intricate host-related immune reaction to the infection. While current literature extensively explores the immune response to VL, dissecting cellular responses in detail (5), the reasons behind the variability in this response among individuals remain elusive and comprehensive review addressing the immune response across the spectrum of clinical manifestations is notably absent.

Through this narrative review, we aimed to shed light on the underlying immunological mechanisms that contribute to the varying spectrum of clinical conditions observed in *Leishmania* infection. Understanding these mechanisms can have significant implications for future research, particularly in the development of individually tailored therapies and preventive strategies. The structure of the article involves a comprehensive analysis of the immune response, starting from distinct clinical manifestations: asymptomatic, symptomatic VL, hemophagocytic lymphohistiocytosis (HLH), and post-kala-azar dermal leishmaniasis (PKDL).

## 2 Methods

In order to perform a narrative review of the available literature, we searched the PubMed and Embase database from March 2018 through March 2023, using the following keywords: immune, immunity, Leishmania, donovani, infantum. The search and the selection process were not systematic. Articles were limited to English language, full text availability, human species and they were excluded if they were redundant or not pertinent. References of all relevant articles were also evaluated, and studies published previously than 2018 were cited if considered relevant. Results were critically summarized in the following paragraphs: (1) Immunity and symptomatic visceral leishmaniasis, (2) Asymptomatic infection of viscerotropic *Leishmania* strains and persistence within the human body, (3) Visceral leishmaniasis-associated hemophagocytic lymphohistiocytosis-mimic and (4) Immunity and post-kala-azar dermal leishmaniasis. The main information from the cited articles has been elaborated and summarized, as shown in the tables within the supplementary material (**Supplementary Tables S1–S4**). This review was conducted in accordance with the SANRA checklist to ensure comprehensive and structured coverage of the subject matter.

In literature, there is no univocal consensus regarding the definition of these clinical manifestations, therefore we propose a nomenclature that will be used hereafter in the present review. The terms active disease and chronic disease were intentionally omitted as they can be misleading.

- Asymptomatic *Leishmania* infection (ALI): asymptomatic individual with no history of unexplained fever who tested positive in at least one assay that confirms the exposure to the parasite. Parasite exposure can be proved by serological testing, Leishmanin skin test (LST) and interferon gamma release assay (IGRA) or the direct detection of the parasite using Polymerase Chain Reaction (PCR).
- Visceral leishmaniasis (VL): symptomatic infection marked by persistent fever, hepatosplenomegaly and mono/multilinear cytopenia in a patient who has tested positive in at least one assay confirming the exposure to the parasite. In the existing literature, this clinical condition is alternatively referred to as kala-azar (derived from the Hindi term), as well as active VL or chronic VL.
- VL-associated hemophagocytic lymphohistiocytosis-mimic: clinical course of VL can be complicated by signs and symptoms that mime HLH disease. HLH is a potentially life-threatening syndrome characterized by uncontrolled activation of the immune system, leading to hyperinflammation and tissue damage caused by a lack of normal downregulation of activated immune cells. According to HLH-2004 guidelines (6), the diagnosis of HLH is performed when at least 5 of the following criteria are fulfilled: 1) fever, 2) splenomegaly, 3) cytopenia affecting at least two of three lineages in the peripheral blood, 4) hypertriglyceridemia and/or hypofibrinogenemia, 5) hemophagocytosis in bone marrow, spleen, or lymph nodes, 6) low or absent NK-cell activity, 7) hyperferritinemia and 8) elevated soluble CD25. HLH is defined as primary when linked to a genetic anomaly or secondary when triggered by any other cause. VL is historically noted as one of the possible infections linked to secondary HLH. However, due to the clinical and laboratory overlap between VL and HLH and, VL-associated HLH as a distinct nosological entity may be called into question and it should be considered more accurate referring to this condition as VL-associated HLH-mimic
- Latent *Leishmania* infection: persistence of viscerotropic strains within the human body following the primary *Leishmania* infection which might have been asymptomatic or resolved through medical treatment. The latent infection can reactivate even years to decades after the primary infection in the event of host-immunosuppression (e.g. HIV infection)
- Post-kala-azar dermal leishmaniasis (PKDL): dermatological manifestation appearing within months to years after successful treatment of VL caused by *L. donovani*.

## 3 Immunity and symptomatic visceral leishmaniasis

In 2022, 13’000 cases of VL were reported worldwide, among which almost 50 occurred in Europe (7). These figures are likely significantly underestimated due to underdiagnosis and underreporting, especially in tropical areas. With a mortality rate as high as 95% when untreated, VL stands as one of the most lethal tropical diseases.

Symptoms usually develop insidiously or subacutely, progressing slowly over weeks to months, and include malaise, fever, weight loss, and splenomegaly, sometimes accompanied by hepatomegaly. Lymphadenopathy is more common in East African VL (8).

High parasite loads accumulate in the spleen, liver, and bone marrow, leading to severe anemia from bone marrow suppression, hemolysis, and splenic sequestration. Late-stage disease can involve hepatic dysfunction, jaundice, ascites and spontaneous bleeding from various sites due to thrombocytopenia and hepatic impairment (8). Chronic diarrhea and malabsorption are rare but possible due to intestinal parasitic invasion (9).

The risk of developing symptomatic VL is influenced by a complex interplay of various factors, encompassing poor socio-economic conditions, parasite-related factors, and host-related factors, first and foremost the immune system (4).

The immune response against viscerotropic *Leishmania* strands starts upon the inoculation of the promastigotes into the host’s skin. The initial defense is carried out by the complement system, which has the ability to lyse nearly 90% of the introduced parasites (10). However, parasites are able to exploit both their own surface metalloproteases and host’s complement regulatory proteins to shield themselves from complement attack (11, 12), This way, *Leishmania* avoids complement mediated lysis and simultaneously takes advantage of the complement coating exploiting it as a bait to facilitate uptake by macrophages, the parasite’s ultimate target (13).

Moreover, the parasites’ inoculum triggers the recruitment of phagocytic cells, primarily neutrophils which typically reach the site of infection within a couple of hours. The pathogen-associated molecular patterns (PAMPs) expressed by *Leishmania* spp are recognized by various toll like receptors (TLRs) expressed mainly by antigen-presenting cells. Previous studies have focused specifically on TLR2 (14) and TLR9 role (15). This recognition triggers a pro-inflammatory cascade, leading to the recruitment and activation of neutrophils, which in turn respond by phagocytosing the parasite. Within neutrophils, *Leishmania* faces two possible fates: oxidative-burst-dependent death or evasion of neutrophil cytotoxic mechanisms. The latter mainly occurs by inhibition of lysosome fusion, thus allowing the parasite to reach and hide within the non-lytic intracellular compartment of neutrophils (16). Interestingly, a study conducted by Sharma et al. revealed that surviving *Leishmania* parasites significantly influence the transcriptional profile of neutrophils, inducing the expression of interleukin-10 (IL-10) and Arginase-1—proteins associated with *Leishmania* persistence, as elaborated later (17). After two days, the infected neutrophils undergo apoptosis and are phagocytosed by macrophages, bringing with them viable and replicating parasites. This invasion strategy, known as the Trojan Horse, allows *Leishmania* strains to enter and proliferate almost unnoticed within macrophages: indeed, the lack of direct contact between the parasites and macrophagic receptors prevents activation toward a pro-inflammatory phenotype (18).

Once macrophages phagocytose the parasite, whether directly or via the Trojan horse mechanism, *Leishmania* promastigotes, having reached their ultimate cellular target, turn into actively replicating amastigotes. At this stage, the parasite employs various strategies to evade lysis induced by parasiticidal agents within the phagolysosome (19) and alter pro-inflammatory cytokines production (20) as brilliantly reviewed by Carneiro et al (21).

These infection-induced alterations not only hinder macrophages’ own independent killing capacity but also affect antigen presentation and T cell activation, deeply influencing the connections between innate and adaptive immunity (22). The containment of infection is compromised by the shift in the inflammatory environment toward an anti-inflammatory (macrophage M2/T helper 2) milieu, more permissive for parasite survival. This fact has been extensively demonstrated since the 1980s when numerous experiments conducted on murine models revealed susceptibility to severe disseminated *L. major* infections in mice with T helper 2 (Th2) prevalence (23).

**Figure 1:**
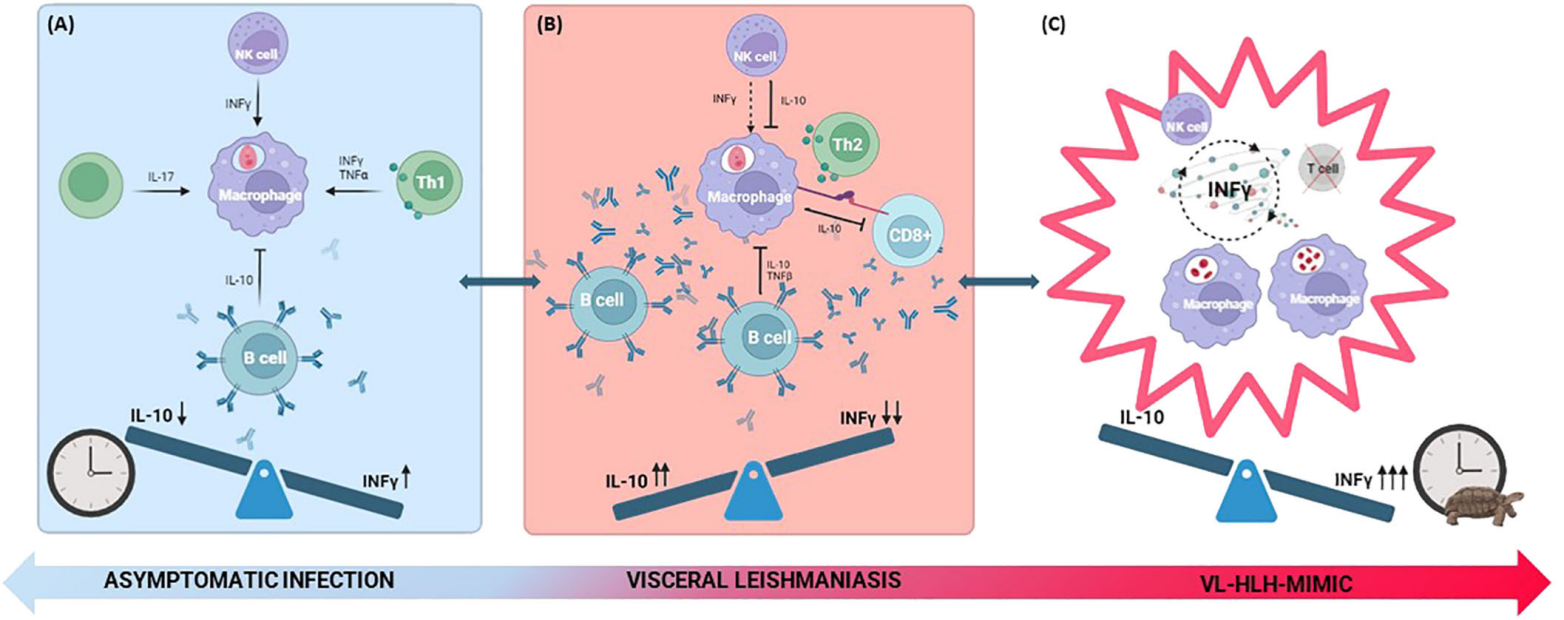
Depiction of cellular immune interplay in effective **(A)**, ineffective **(B)** and frustrated **(C)** response. **(A)** The parasites induce a pro-inflammatory response, where INF-γ is predominant. The response is mediated by CD4+ T cells, T helper 17 and NK cells secreting pro-inflammatory cytokines (INF- γ, TNF-α and IL-17). B cells play a minor role producing non-protective antibodies and secreting anti-inflammatory mediators such as IL-10. **(B)** The response is characterized by an anti-inflammatory environment dominated by a Th2 response, where IL-10 becomes predominant. Infected macrophages express PDL-1 and secrete IL-10, contributing to the exhaustion of cytotoxic CD8+ T cells. NK cells and CD4+ T cells produce IL-10, while B cells play a more central role causing a marked hypergammaglobulinemia associated with the production of polyclonal non-protective antibodies, downregulating defensive pro-inflammatory mechanisms. **(C)** The exhaustion of T lymphocytes and persistent immune stimulation result in unchecked proliferation and phagocytic activity of macrophages. This cycle is further fueled by INF-γ released by NK cells, culminating in a cytokine storm. The clock image represents the timeliness of the immune response. The inappropriate timing of inflammatory cytokine production is graphically represented by the clock with the turtle. IL, interleukin; INF-γ, interferon-γ; NK, natural killer; PDL-1, programmed cell death-ligand 1; Th2, T helper 2; TNF-α, tumor necrosis factor-α.

Moreover, infected-macrophages express anti-inflammatory cytokines (such as IL-10) and surface programmed cell death-ligand 1 (PDL-1), an immune checkpoint downregulating T cells response, contributing to T cells exhaustion (24). This exhaustion, demonstrated by an experiment conducted by Habiba et al., is characterized by a shift to a profile of diminished cytokine secretion 21 days after the infection following the initial pro-inflammatory T cell response (CD4+ and T helper 17 (Th17) T cells secreting interferon-γ (IFN-γ), Tumor Necrosis Factor-α (TNF-α) and IL-17), as shown in **Figure 1**. Epidemiological studies in VL patients supported these findings, indicating reduced CD4+ and CD8+ T cells’ expression of pro-inflammatory cytokines even after mitogen stimulation, with restoration of a normal secretion upon antimicrobial therapy (25).

Interestingly, the plasma of VL patients exhibited high levels of IFN-γ and IL-10. Considering that INF-γ could not be secreted by exhausted T cells, the authors postulated that it originated from natural killer (NK) cells, a hypothesis later supported by Prajeeth et al. (26), who elucidated the ability of NK cells to induce INF-γ-associated cytotoxicity. The reason why macrophages and CD4+ T cells do not respond adequately to a pro-inflammatory stimulus like INF-γ remains unknown. Eventually, this phenomenon could be dependent on the high levels of IL-10 (27) secreted by B cells (28) and causing macrophagic cytokines deactivation thus blunting INF-γ activity. Indeed, even NK cells and CD4+ T cells will become important IL-10 producers in the later stages of the infection as a consequence of persistent and extensive activation (29). After pharmacological treatment it is possible to observe a drop in both IL-10 and INF-γ expression, concomitant to the containment of the infection (28).

Regarding the B cells compartment, VL induces a strong B cell activation characterized by hypergammaglobulinemia. These polyclonal low-affinity antibodies are non-protective as they do not contribute to the containment of the infection while they mediate pathology through downregulation of defensive pro-inflammatory mechanisms and induction of autoimmune manifestations (30) and immune complexes. A study conducted by Silva-Barrios et al. revealed that the absence of hypermutated and class-switched antibodies was associated with disease resistance, correlated to a stronger Th1 response and reduced IL-10 production (28).

### 3.1 Visceral leishmaniasis in the immunocompromised host

Immunocompromised individuals, whether due to inborn errors of immunity (IEI) or acquired immunodeficiencies, exhibit heightened susceptibility to symptomatic and potentially life-threatening *Leishmania* infections.

It is not surprising that Human Immunodeficiency Virus (HIV) co-infection and Acquired Immunodeficiency Syndrome (AIDS) is a major susceptibility factor to leishmaniasis, being both HIV and *Leishmania* endemic in many tropical and subtropical areas (31). Worldwide, Ethiopia has the highest rate of VL-HIV co-infections, with 30% of VL cases presenting HIV (32). Although most VL-HIV coinfected patients present typical VL symptoms, there is a notable tendency to involve organs not typically parasitized, such as the lungs, skin and gastrointestinal tract (33–35). This can lead to misdiagnose it as another opportunistic infection, delaying VL diagnosis and contributing to a poor outcome. Indeed, a retrospective cohort study conducted in Brazil found that coinfected individuals had a double relapse rate compared to those without coinfection and the fatality rate among coinfected cases was three times higher than non-coinfected cases (36).

Several authors reported a higher mortality rate in coinfected patients and the cause of death is attributed to additional conditions linked to AIDS, including opportunistic diseases and treatment-related complications (35, 37, 38). Nevertheless, the parasite itself plays a pivotal role in this process, due to the chronic immunostimulation amplifying HIV replication, leading to the advancement of HIV infection toward AIDS (39, 40). Indeed, as shown in a cross-sectional study, in the infected cells *Leishmania* induces an increased expression of C-C chemokine receptor type 5 (CCR5), a co-receptor for HIV entry into CD4+ and CD8+ T lymphocytes, facilitating the increase in HIV viral load and the progression of immunosuppression (41). Also, HIV co-infected patients may present an altered equilibrium between the regulatory T cells (Treg) and immune-activation environment, with an up-regulated expression of Cytotoxic T-Lymphocyte Antigen 4 (CTLA-4) on the autologous Treg, increasing their suppressive function and leading to parasite persistence and higher relapse rate, despite successful HIV and leishmaniasis treatment (41).

As in co-infected patient, also non-HIV immunosuppressive conditions resulting from iatrogenic factors including immune-ablative chemotherapy or immunosuppressive drugs can hinder the immunological control of VL, leading to reactivation from latent infection or inability to control new infection (42). These conditions are frequently seen among transplant, rheumatologic or oncologic patients, as they can affect multiple layers of the immune system, primarily impacting T-cell lymphocytes through mechanisms such as depletion, interference with maturation, cell cycling, co-stimulation, and induction of tolerance (43). Evidence is still lacking for VL screening in immunosuppressive therapy or organ transplantation candidates in endemic areas, and recommendations for secondary prophylaxis in non-HIV immunosuppressed VL patients remain unclear, despite some studies indicating reduced relapses (43, 44).

When HIV co-infection or other causes of acquired immunodeficiency are excluded and a VL patient presents with atypical symptoms, it is mandatory to rule out any kind of IEI. Altered T-cell function, impaired phagocytosis, and deficiencies in cytokine production may contribute to prolonged parasite survival and dissemination in the host. There are few reports in literature of IEI associated with leishmaniasis and in most cases the infection itself was the trigger revealing the primary immunodeficiency. Finocchi et al. were the first to document a case of VL infection in a 3-year-old child with chronic granulomatous disease (CGD) and a poor response to amphotericin B (45). Moreover, Al Ayed et al. reported a case of a 6-month-old infant with disseminated *L. donovani* and CGD. Due to the atypical cutaneous diffusion of the parasite, an immune work out was performed and CGD was confirmed (46). Carvalho et al. described two cases of VL in two different immunocompromised patients, one with GATA2 deficiency and the other one with Griscelli Syndrome, both with relapses after VL treatment, requiring secondary prophylaxis (47).

Late detection of immunodeficiency in atypical VL cases results in delayed diagnosis, prolonged infection, and increased severity. Unraveling genetic susceptibility or other acquired immunodeficiency is crucial for targeted interventions and advancing understanding of host-parasite interactions. As regards the management of immunocompromised patients, currently WHO guidelines are available only for the treatment of VL in HIV co-infected patients. According to a randomized trial conducted in Ethiopia, combination therapy with Liposomal Amphotericin B plus miltefosine is recommended, in place of monotherapy (48). Furthermore, a secondary prophylaxis with pentamidine should be considered after the first episode of VL in patients with CD4+ < 200 cells/μL, as few reports showed a lower relapse-free survival rate compared to patients out of prophylaxis (49, 50).

WHAT WE ALREADY KNEW: The containment of infection is compromised by the shift in the inflammatory environment toward an anti-inflammatory (M2/Th2) milieu, more permissive for parasite survival. Immunocompromised individuals are more susceptible to *Leishmania* infection, which can have an atypical and worse course than in the immunocompetent people. HIV co-infection is very common, due to the similar epidemic area of both virus and *Leishmania*.

WHAT WE NOW KNOW: *Leishmania* parasites hijack the pro-inflammatory cascades exploiting various mechanisms including immune checkpoints. The susceptibility to the infection is strongly dependent upon the finely tuned balance between IL-10 and INF-γ expression. HIV-VL coinfected patients have higher relapse and mortality rate than the one with VL only. Primary prophylaxis is not recommended, but secondary prophylaxis should be started in HIV-VL coinfected patients with CD4 cells count below than 200 cells/μL. VL patients should be screened for primary immunodeficiency, particularly in cases of VL relapse.

WHAT WE STILL DO NOT KNOW: The factors, both host and parasite-related, polarizing the immune response toward an M2/Th2 profile in individuals who develop a symptomatic infection are still not known. The indications for secondary prophylaxis remain to be defined in non-HIV-related immunocompromised patients.

## 4 Asymptomatic infection of viscerotropic *Leishmania* strains and persistence within the human body

Quantifying the exact prevalence of asymptomatic *Leishmania* infection is extremely challenging as it would imply the screening of healthy subjects in endemic tropical areas. Adding to the complexity, the lack of a clear consensus on the definition of asymptomatic patients leads to substantial variability in the diagnostic tests used across studies, such as LST, serological analysis, or PCR.

A systematic review and meta-analysis estimated that the prevalence of *L. infantum* and *L. donovani* infections in otherwise healthy individuals in endemic areas were 13.4% and 6.9% respectively. Consequently, a striking 68% of all viscerotropic *Leishmania* infections were asymptomatic (51). In a Northen region of Italy, according to a study conducted by Ortalli et al. the estimated prevalence of *L. infantum* infection among asymptomatic blood donors was reported to be 12.5% (52).

It would follow that most individuals who encounter viscerotropic strains of *Leishmania* are able to mount an immune response able to contain and eradicate the infection, without even exhibiting the related symptoms. The idea that a parasite, which can potentially be lethal, is in fact largely harmless to its host in the majority of cases, casts doubt on its inherent pathogenicity and suggests that susceptibility to this infection may be due to one’s immunological profile. However, it is important to underline that among the population of asymptomatic carriers, there might be a subgroup of individuals who will later develop symptoms. This subgroup could include patients categorized as asymptomatic because they were tested during their lengthy incubation period. A prospective cohort study by Chakravarty et al. found that 8 out of 476 seropositive patients developed VL during a 3-year follow-up (53). Numerous studies have proposed various biomarkers, including cytokine profiles, antibody titers, and antibody avidity, as potential indicators to differentiate and even predict the progression of the infection to symptomatic disease (53–56).

Moreover, it is now widely recognized that viscerotropic *Leishmania* strains have the ability to persist within the human body even after medical treatment or self-healing, possibly leading to a subsequent reactivation of the disease, even in cases in which the primary infection has progressed asymptomatically. The notion of the persistence of *Leishmania* parasites in asymptomatic patients’ questions whether the human body is able to eradicate the infection at all. At present, the confirmation of parasite persistence is primarily established upon disease relapse, in the form of PKDL or VL reactivation during periods of immunosuppression (i.e. in cases of HIV infection). Currently, there is no evidence on the possibility of reactivation in immunocompetent patients. A study conducted in Ethiopia revealed that after a 3-years follow-up, 78.1% of VL-HIV coinfected patients experienced relapses as opposed to the cohort of immunocompetent VL patients who did not experience any relapse (57).

A combination of host related factors such as nutritional status, socio-economic conditions, and genetic factors play a crucial role in the manifestation of asymptomatic infections. Regarding age-specific factors, it’s interesting to note that both children and elderly people may present with asymptomatic infections. Indeed, according to the systemic review and meta-analysis conducted by Mannan et al. the prevalence of asymptomatic leishmaniasis in children residing in endemic areas is 10.9% (51). Genome wide association studies have shed light on specific genetic traits, such as HLA-DRB1*15 and HLA-DRB1*16, which are associated with a lower susceptibility to VL (58, 59). Furthermore, despite HIV-infected patients being more susceptible to severe forms of VL, some individuals within this cohort may still develop asymptomatic infections [up to 9% in an endemic area of India (60)], underlining the complexity and variability of the immune response to *Leishmania* in different individuals. The asymptomatic HIV-infected patients were younger, more often on antiretroviral therapy and with a higher CD4+ count compared to the symptomatic group (61).

Giorgio et al. have postulated that the discriminating factor between an effective immune response and an ineffective one is one’s ability to form granulomas able to contain the parasite (62). This theory is based on histological findings discovered during an early study highlighting the presence of granulomas on liver biopsies in asymptomatic infected patients (63). Indeed, asymptomatic patients exhibit a pro-inflammatory macrophage (M1) and T helper cells polarization (Th1) response, mainly mediated by CD4+ T cells, CD8+ T cells, and invariant NKT cells (61, 64, 65). This pro-inflammatory milieu is prompted by neutrophils which will prime and shape the following macrophage leishmanicidal response (5, 66). Macrophages, despite being the primary target for the parasites, play a crucial role in the containment of the infection by both mediating the killing of parasites and inducing an effective T cell response. Likewise, NK cells contribute to a pro-inflammatory state by stimulating the production of IFN-γ (26, 67).

In theory, the more the immune response is skewed toward a pro-inflammatory M1/Th1 response, the higher the likelihood of effectively containing the parasite. Furthermore, this theory is corroborated by the presence of Th17, highly pro-inflammatory helper T cells, in asymptomatic and healing VL patients, whereas their expression is downregulated in VL symptomatic patients (68). Nevertheless, when analyzing the distribution of CD4+ T cell subsets in asymptomatic hosts, a notable shift toward both Th1 and T regulatory subsets can be observed (64). This observation underscores the intricate balance between creating a pro-inflammatory environment that is essential for containing the parasites and concurrently preventing immune-mediated tissue damage.

In order to establish a latent infection, that parasites can withstand the attempts of the immune system to clear them, leading to the establishment of a persistent infection through two main mechanisms:

1. by enduring the pro-inflammatory (M1) environment; *Leishmania* parasites can persist within macrophages, dendritic cells, reticular fibroblasts and long-term hematopoietic stem cells (69), displaying resilience to the toxic activity of phagocytic cells’ oxidative stress. Mandell and Beverley (70) demonstrated that *Leishmania* parasites can both survive and actively replicate within these cells. In their *in vitro* and murine models, the overall number of persistent parasites remained relatively constant over time, suggesting that ongoing destruction of some parasites must also be occurring at the same time, thus underlying the finely tuned equilibrium between the parasite and the host immune system, ultimately peacefully coexisting. In theory, the active proliferation of the parasite provides the continuous antigenic stimulus preventing a second *Leishmania* infection: this concept is known as concomitant immunity. However, in their experiment, the persistence of *L. major* did not impede super-infection with a second *L. major* line even though it conferred an advantage in resulting in less severe clinical pathology (71). This result has a very important implication in the context of vaccine development. Indeed, future vaccines aiming at inducing the persistence of live-attenuated parasites could not prevent supra-infections with other *Leishmania* parasites.
2. by finding refuge within niches where the anti-inflammatory (M2) environment prevails. Interestingly, the mutual influence between macrophages and viscerotropic *Leishmania* strains alters the inflammatory milieu found within granulomas. Indeed, some authors hypothesized the presence of niches within the granulomas characterized by a Th2/M2 environment permissive to amastigote persistence and active proliferation inside the macrophages. These niches could serve as a parasitic reservoir resistant to medical treatment, potentially leading to reactivation of the infection during immunocompromised status (62, 72). Notably, this finding seems to be more creditable for spleen granulomas while liver granulomas can contain the parasite more effectively by showing a more inflammatory environment. This theory is corroborated by the fact that murine and hamster models infected with *L. donovani* were found to express in the spleen higher levels of Arginase 1, an enzyme associated with Th2 environment and persistence of *Leishmania* (73–75).

Moreover, persistent antigenic stimulus may lead to T cells exhaustion, as previously illustrated.

The notion of *Leishmania* persistence within the human body leads to several implications in a clinical setting. The most important one being the necessity of clinically monitoring all immunosuppressed patients who encountered viscerotropic strains of *Leishmania* during their lifetime, given our current difficulties in discerning those who successfully eradicated the disease from those still harboring actively replicating parasites. Takele et al. proposed the utilization of RNA sequencing to measure L. donovani mRNA in blood as a valuable tool to monitor parasitic load over time (57). Another crucial consideration is the need to establish guidelines for secondary prophylaxis. Currently, according to the Infectious Disease Society of America (IDSA) guidelines, chronic maintenance therapy (secondary prophylaxis) is only indicated in case of HIV-coinfection and low CD4+ counts. In all other instances, treatment is limited to patients with evidence of clinical and parasitological relapse (76).

WHAT WE ALREADY KNEW: most individuals encountering viscerotropic *Leishmania* strains are able to mount an effective immune response ultimately leading to an asymptomatic course of the infection. VL strains can persist within the human body after spontaneous or iatrogenic recovery and reactivate in the form of PKDL or VL reactivation during periods of immunosuppression (e.g. during HIV infection). The persistence of the parasite can induce concomitant immunity thus impeding a second clinically severe VL, even though it most probably cannot prevent a secondary *Leishmania* infection.

WHAT WE NOW KNOW: approximately 68% of viscerotropic *Leishmania* infections will have an asymptomatic course. The effective infection-containing immune response is mainly based on pro-inflammatory Th1/M1 response leading to the formation of granulomas. VL strains can both resist the immunity attack and persist in various niches within the body. The persistence of the parasite does not prevent colonization by *Leishmania* strains even though it diminishes clinical pathology, provided that the immune system is still competent.

WHAT WE STILL DO NOT KNOW: we are still lacking a clear and universally accepted definition of asymptomatic *Leishmania* infection along with suitable diagnostic tools, also due the difficulties tied to the extended incubation period associated with this condition. The prevalence of persistent viscerotropic *Leishmania* infection in humans, its duration and its transmission risk are still not known.

## 5 Visceral leishmaniasis-associated hemophagocytic lymphohistiocytosis-mimic

Leishmaniasis was historically recognized as one of the primary protozoan infections capable of triggering secondary HLH, a life-threatening syndrome characterized by a maladaptive immune response involving T cells and innate immunity (77, 78). HLH was traditionally defined as secondary when a precise genetic predisposition could not be identified and a patent environmental trigger (infectious, iatrogenic, malignant, etc.) was present. However, with the increasing understanding of the genetic complexity underlying HLH the dichotomy between primary (genetically defined) and secondary (environmentally triggered) appears to be oversimplifying and misleading (79). In this regard, the status of VL-associated HLH (VL-HLH) as a distinct nosological entity can be subject to questioning, due to the extensive overlap in clinical features between VL and HLH (80). Indeed, it is plausible that individuals previously diagnosed with VL-HLH actually exhibited just a severe form of symptomatic VL.

According to the HLH-2004 trial, HLH diagnosis is established if at least five of the subsequent criteria are met: fever, splenomegaly, bilinear cytopenia, hypertriglyceridemia and/or hypofibrinogenemia, tissue hemophagocytosis phenomena, reduced NK-cell activity, hyperferritinemia, and elevated sCD25 (sIL-2R) levels (6). Since the most common clinical manifestations of VL include fever, hepatosplenomegaly and mono/multilinear cytopenia, it is evident that *Leishmania* infection itself can emulate the hemophagocytic syndrome, making their differentiation exceedingly challenging. Rajagopala et al. in their review of patients with VL and HLH reported the following findings: patients presented with fever (100%), splenomegaly (98%), hepatomegaly (80%), pancytopenia (88.9%) and lymphadenopathy (11%) (81). These signs and symptoms are non-specific to a single entity and may manifest in either VL, HLH, or both conditions simultaneously (4, 6).

Regarding laboratory findings, hyperferritinemia is another characteristic feature of VL, associated with systemic inflammation, along with elevated levels of IL-2 indicating T-cell activation (82). Furthermore Chandra et al., in their clinical study examining morphological features through bone marrow aspirate cytology, demonstrated mild to moderate hemophagocytosis in 70.3% of cases, indicating that this finding may occur in VL as a component of dyserythropoiesis (83).

In addition to consolidated HLH biomarkers encoded by the 2004 diagnostic criteria, some authors have identified an expansion of CD38^high^/HLA-DR^+^CD8^+^ T cells in HLH (84, 85), that relates to an increase of sIL-2R as an expression of T cell activation (86) which has also been described in forms classified as VL-HLH (87). However, this lymphocyte population seems to show different degrees of expansion in all forms of VL which would make it unsuitable for discriminating between VL and HLH (unpublished data).

To further cast doubt on the existence of VL-HLH as a distinct entity is the response to different treatments, as VL-HLH consistently responds to VL antimicrobial therapy (88, 89) whereas in full-blown HLH triggered by infections such as EBV, mere infection control without immunomodulatory therapy can be insufficient for HLH control (90).

In Europe and the United States, liposomal amphotericin B (L-AmB) is recommended as first-line treatment for VL. Moreover, its efficacy in VL-HLH patients was extensively demonstrated (88) (89). Further immunosuppressive therapy as dictated by the HLH-94/04 protocol or novel immunomodulatory approaches as JAK-inhibithors or emapalumab could be unnecessary or even dangerous in VL-HLH as treatment with anti-parasitic drugs alone is sufficient for complete recovery (81) (91–93). Moreover, there were no differences in terms of outcome and relapses between two cohorts of patients diagnosed with VL and HLH, the first one treated with L-AmB monotherapy and the other one with L-AmB and additional immunomodulatory therapy with corticosteroids (94). Indeed, in their Recommendations for the Management of HLH in Adults, La Rosée et al. advise against immunosuppressive therapy (95). Nevertheless, several authors proposed that in more severe or refractory cases, corticosteroids treatment could be beneficial to cease the cytokine storm (78, 96).

With these premises, however, considering the historical significance in literature of the relationship between VL and HLH, the topic will be further addressed in this paragraph referring to this entity as VL-associated HLH-mimic.

A retrospective cohort study conducted by Daher et al. revealed that 27.6% of Brazilian children with VL developed HLH during the disease’s progression (97). This percentage rose to as high as 41.7% in a multicentric case series conducted in Spain (98).

While the pathophysiology of HLH has been extensively studied, the precise mechanisms potentially triggering HLH in the context of VL remain incompletely understood. Indeed, the chronic and persistent nature of the infection can lead to a sustained immune activation and dysregulation, impairing the ability of NK cells and cytotoxic T lymphocytes to limit activated phagocytic cells (99–102). The unchecked activation of macrophages will ultimately lead to the phagocytosis of the host’s cells (103).

One of the main mechanisms underlying the pathogenesis of this condition is the significantly skewed production of cytokines toward a pro-inflammatory state, the so-called cytokine storm. As shown in a retrospective study in Beijing, Th1 cytokines such as IFN-γ were found to be higher in VL-HLH patients compared to those with VL alone where the Th2 profile seems to prevail (104). The cytokine storm reinforces the vicious cycle of NK cells and macrophages’ activation ultimately resulting in hemophagocytosis, tissue damage, organ failure and other inflammatory manifestations (105). It could be speculated that this could happen as a result of an initial ineffective immune response that leads to parasite persistence, prolonged immune stimulation and overly late skew toward a Th1 profile, which ultimately results ineffective for parasite control in that phase and initiates the HLH-mimic cycle, as illustrated in **Figure 1**.

Although VL-associated HLH-mimic is considered a rare complication in immunocompetent adults, it is frequently seen in immunocompromised adults and children, especially under 5 years of age (106–108). Specifically, in a retrospective study conducted in Spain, it was noted that within the pediatric population, children under two years of age were more susceptible to this complication. Therefore, Lopez et al. hypothesized that the immaturity of the developing immune system puts younger children at an elevated risk of developing HLH (94). Furthermore, in patients with impaired function of phagocytic cells, associated with IEI such as CGD, the risk of HLH triggered by *Leishmania* infection seems to be increased (109). The very fact that patients more susceptible to severe course of the infection are more prone to develop VL-HLH corroborates the thesis that these two clinical manifestations may be a continuum of the same spectrum.

Likewise, due to the strong association between these two clinical entities, a patient presenting with HLH in an endemic region should always be investigated for VL and VL-associated HLH-mimics cases investigated for underlying IEI. Indeed, two significant studies conducted in Spain and in Southern Iran demonstrated that VL was responsible for 20% and 33.3% of the analyzed HLH cases, respectively (78, 88). These percentages exceeded what has been reported in the existing literature, suggesting that this association might have been previously underestimated or that the prior diagnosis of HLH may have been incorrect.

Furthermore, as indicated by a retrospective study conducted in Germany involving a pediatric cohort of HLH and VL patients, the global migratory patterns and the impact of climate change are modifying the distribution of VL, extending it beyond tropical regions (89). As a result, applying a systematic evaluation for potential *Leishmania* infection should be a part of the routine diagnostic work-up in patients with HLH, even in regions that are not traditionally endemic for *Leishmania*. This proactive approach helps to prevent unnecessary and potentially detrimental high-dose immunosuppression, which would not address the underlying infectious cause of the immune dysregulation.

WHAT WE KNEW: Historically, *Leishmania* has been considered a triggering pathogen for HLH and the clinical course of VL can be complicated by HLH, especially in the pediatric population. In VL-HLH, antimicrobial therapy of the underlying cause may be sufficient for complete recovery.

WHAT WE NOW KNOW: Currently, the existence of the nosological entity VL-HLH is debated due to the clinical and laboratory overlap between the two conditions and it should be considered more accurate referring to this condition as VL-associated HLH-mimic. It is essential to exclude VL in patients presenting with HLH. Due to the spread of viscerotropic *Leishmania* strains in traditionally non-endemic regions, it is important to screen for *Leishmania* also patients presenting with HLH in non-endemic regions. Patients presenting with VL-associated HLH-mimic should undergo a complete immune work-up to rule out underlying IEI like CGD.

WHAT WE STILL DO NOT KNOW: Unraveling shared pathogenic mechanisms between severe forms of VL and HLH not associated with leishmaniasis can provide insights into the functioning of HLH and facilitate the identification of potential new therapeutic targets. The role of systemic corticosteroid therapy in severe forms of VL-associated HLH-mimic might be beneficial but is still debated.

## 6 Immunity and post-kala-azar dermal leishmaniasis

PKDL is a dermatological complication occurring months to years after successful treatment of *L. donovani*-associated VL (110).

PKDL is prevalent in East Africa and the Indian subcontinent, where *L. donovani* is endemic, emerging in 5-15% of South Asian patients within 5 years and up to 50% in East Africa within months (110, 111). Interestingly, 16% of Sudanese patients presented PKDL lesions during VL treatment, suggesting a potential new clinical entity called para-kala-azar dermal leishmaniasis (112).

Furthermore, the clinical presentation varies as shown in **Table 1**. African patients present papular, macular or nodular lesions mostly healing spontaneously within 6 months to 1 year from onset, whereas in South Asia, PKDL is not self-limiting and presents in two distinct clinical forms, the polymorphic or the macular one (113).

**Table 1:** Comparison of most important features of African and Asian PKDL.

	East Africa	Southern Asia
Epidemiology		
Incidence of PKDL after VL	50-60%	5-15%
Interval between VL and PKDL	0-6 months	2-3 years (until 30 years)
Clinical		
PKDL with absent evidence of previous VL	Yes (10% of cases)	Yes
PKDL with evidence of previous VL	Yes (60% of cases)	Yes
Para-kala-azar form	Frequent (15% of cases)	Rare
Body distribution	Face>trunk>arms>legs (usually symmetrical)	Face>trunk>arms>legs
Type of skin lesions	Papulo-nodular > Maculo-papular > Micro-papular > Macular	Polymorphic (both macules and indurated lesions such as papules are present) > monomorphic (macular or nodular) > other forms (e.g. erythrodermic form)
Grades	Grade 1 – scattered maculopapular or nodular rash on the face, with or without lesions on the upper chest or arms;	Mild (very few lesions, usually on the face);
	Grade 2 – dense maculopapular or nodular rash covering most of the face and extending to the chest, back, upper arms and legs, with only scattered lesions on the forearms and legs;	Moderate (lesions easily visible and generalized);
	Grade 3 – dense maculopapular or nodular rash covering most of the body, including the hands and feet; the mucosa of the lips and palate may be involved.	Severe (dense coverage with lesions and little normal skin remains).
Treatment		
Self-healing condition	Yes, 85% of cases heal spontaneously within 1 year.	Not reported, all cases are treated
Treatment regimens	• Sodium stibogluconate (20 mg/kg Sb5+ per day) for 30–60 days *or*	• Amphotericin B deoxycholate: 1 mg/kg per day by infusion, up to 60–80 doses delivered over 4 months or
	• Combination of paromomycin (11 mg/kg base per day) for 17 days plus sodium stibogluconate (20 mg/kg per day) for 17–60 days *or*	• Miltefosine: 100 mg orally per day for 12 weeks for patients weighing >25 kg; 50 mg orally per day for 12 weeks for patients weighing <25 kg
	• Liposomal amphotericin B (2.5 mg/kg per day) for 20 days when indicated	• Liposomal amphotericin B: 5 mg/kg per day by infusion two times per week for 3 weeks for a total dose of 30 mg/kg
	• Miltefosine: 100 mg per day for 28 days may be beneficial in patients coinfected with HIV and PKDL	

PKDL, Post Kala-azar dermal reaction; VL, Visceral Leishmaniasis.

PKDL poses a significant public health concern increasing the spread of VL: indeed, patients’ skin lesions act as a reservoir of viable parasites (114–117).

To date, the reasons why *L. donovani* turns its tropism from visceral to dermal during a reactivation is still debated. Several factors, including treatment regimens, environmental conditions, host immune system, as well as parasite-related factors, are proposed to influence this transition from cured VL to PKDL.

Since PKDL lesions typically appear on sun-exposed-skin (face, ears, arms) it is believed that UV light plays a crucial role in PKDL susceptibility (112, 118). Indeed, UV light, especially UVB, is a potent immunosuppressive agent causing a dysfunction in the antigen-presenting activity of epidermal Langherans cells, producing cellular morphology alterations such as shortening and loss of dendrites, swelling and rounding, that ultimately lead to a reduction of expression of the major histocompatibility complex (MHC) class II (118). Moreover UV light plays a role in the generation of Treg cells and leads to skewed cytokine production (119).

Host immune response has a decisive role in PKDL development. As broadly demonstrated, after antileishmanial treatment for VL the immune response shifts to Th1 dominance, with increased IL-12 and INF-γ production, leading to an efficient response to the parasite (120).

On the other hand, in PKDL patients there is a discrepancy in the immune response observed between the skin and the visceral organs. Indeed, as reported by Mukhopadhyay et al, PKDL patients present a systemic Th1 response after treatment, with a deviation toward Th2 and Treg response at a cutaneous level, promoting M2 macrophage polarization, exerting a pro-leishmania activity (121). It could be speculated that the parasite evades treatment by concealing itself within the skin cells where the environment is more permissive.

Mukherjee et al.’s analysis of histopathological data from intra-lesional PKDL biopsies confirmed the predominance of a Th2 environment (122). Notably, these biopsies exhibited a diffuse dermal inflammatory infiltrate primarily composed of lymphocytes and macrophages. The extent of the infiltrate varied, ranging from severe involvement in the polymorphic form to a patchy perivascular distribution in the macular variant (113, 122). It is worth noting that Indian PKDL lesions displayed a significant increase in intra-lesional CD8+ T-cells, along with an almost complete absence of CD4+ T-cells. CD8+ T-cell cytotoxicity activates the NLRP3 inflammasome, causing the release of IL-1β and contributing to chronic inflammation (113). On the other hand, African PKDL presented a predominance of CD4+ T-cells and an overall reduction of the inflammatory infiltrate, explaining the relatively milder presentation and the possibility to self-heal (123).

Elevated IL-10 levels in both the skin and plasma could serve as a predictive factor for the development and severity of PKDL, according to findings from a Sudanese study (124).

HIV co-infection is another risk factor for PKDL development and its severity, especially in patients with low CD4+ counts (125). In most cases skin lesions appear during VL treatment, as in a para-kala-azar form, and they are more widespread, with mucosal involvement also being more frequently observed. Furthermore, PKDL in co-infected patients is not restricted to *L. donovani* as cases of *L. infantum* have been reported from the Mediterranean area as well as from Latin America (126).

Regarding the genetic host susceptibility, some studies have shown an association between PKDL and a specific polymorphism of IFNγ receptor, which results in decreased responsiveness to the IFNγ allowing parasite survival and growth (127). Additionally, the parasite genetic profile seems to be determinant in the clinical manifestations. Dey et al. identified different polymorphisms in a well-defined genetic locus (b-tubulin) of *L. donovani* DNA, analyzed from VL and PKDL patients. The results showed three different recurrent patterns among the VL strains and a genetic homogeneity in all PKDL isolates, suggesting the importance of this specific genetic locus in determining skin or visceral involvement (128).

The treatment of PKDL is essential to alleviate symptoms but also to prevent the transmission. Treatment choice depends on the regional diversity and available resources, according to the World’s Health Organization (**Table 1**) (129).

Challenges in PKDL management arise from limited understanding of its pathogenesis and the role of host immunity. Comprehensive research is essential for effective prevention, and efforts to control PKDL must be integrated into leishmaniasis control programs to achieve sustainable progress in reducing the global burden of this debilitating condition.

WHAT WE KNEW: PKDL is a dermatological complication post-treatment of *L. donovani*-associated VL. The patients’ skin lesions represent a reservoir of the parasite, contributing to its transmission.

WHAT WE NOW KNOW: African and Indian PKDL patients have different characteristics, both for the clinical presentation and the healing process. It seems that UV light exposure increases the susceptibility for PKDL. In PKDL patients, the host immune system presents a Th2 and Treg response, with a M2 macrophage polarization, which creates a pro-parasite environment. In HIV co-infected patients, PKDL can occur also in patients with *L. infantum* infection. A new clinical entity known as para-kala-azar is characterized by the presentation of typical PKDL lesions during VL treatment.

WHAT WE STILL DO NOT KNOW: the exact pathogenetic mechanism underlying the onset of PKDL and how to prevent it is still unknown. The reason why in the skin there is a Th1 failure after VL treatment, with a persistence of Th2 immune response is still debated. Similarly, questions persist regarding whether in PKDL manifestation, the parasite migrates from viscera to seek refuge in the skin, if it represents a reactivation of parasites latent in skin cells, or if it signifies reinfection with cutaneous localization.

## 7 Conclusions

VL is a severe and traditionally tropical disease with high mortality rates, now extending its impact to other regions such as central Europe due to climate change and migratory flows (130). A comprehensive understanding of the immune-host interaction and the interpersonal differences underlying *Leishmania* infection is crucial to guide research in identifying specific markers and predictive models to foresee the disease course. This knowledge could lead to new tailored therapeutic approaches and more effective preventive strategies, in order to reach the ultimate goal of disease burden control.

## References

[1] Jimenez-Marco T Fisa R Girona-Llobera E Cancino-Faure B Tomás-Pérez M Berenguer D. Transfusion-transmitted leishmaniasis: A practical review. Transfusion. (2016) 56(Suppl 1):S45–51. doi: 10.1111/trf.13344 27001361

[2] Dantas Brito M Campilho F Branca R Pinho Vaz C Silva C Sousa T. Visceral leishmaniasis: A differential diagnosis to remember after bone marrow transplantation, case rep. Hematol. (2014) 2014:587912. doi: 10.1155/2014/587912 PMC4276680 25574404

[3] Galindo-Sevilla N Mancilla-Ramírez J. T-cell tolerance as a potential effect of congenital leishmaniasis on offspring immunity. Parasite Immunol. (2019) 41:1–7. doi: 10.1111/pim.12540 29888463

[4] WHO. Leishmaniasis. (2023). Vl, 1–5. Available online at: https://www.who.int/news-room/fact-sheets/detail/leishmaniasis (Accessed February 1, 2024).

[5] Saha B Bhattacharjee S Sarkar A Bhor R Pai K Bodhale N. Conundrums in leishmaniasis. Cytokine. (2020) 145:155304. doi: 10.1016/j.cyto.2020.155304 33004260

[6] Henter JI Horne A Aricó M Egeler RM Filipovich AH Imashuku S. HLH-2004: Diagnostic and therapeutic guidelines for hemophagocytic lymphohistiocytosis. Pediatr Blood Cancer. (2007) 48:124–31. doi: 10.1002/pbc.21039 16937360

[7] WHO. Leishmaniasis, number of cases of visceral leishmaniasis reported - World Health Organization (2021). Available online at: https://apps.who.int/neglected_diseases/ntddata/leishmaniasis/leishmaniasis.html?geog=0&indicator=i4&date=2022&bbox=-37230.90869764579,1765437.950907355,-37199.856282507266,1765448.3517162902&printmode=true (Accessed February 1, 2024).

[8] CDC. Clinical Overview of Leishmaniasis (2024). Available online at: https://www.cdc.gov/leishmaniasis/hcp/clinical-overview/index.html#cdc_clinical_overview_clin_fea-clinical-features (Accessed February 1, 2024).

[9] Baba CS Makharia GK Mathur P Ray R Gupta SD Samantaray JC. Chronic diarrhea and malabsorption caused by Leishmania donovani. Indian J Gastroenterol. (2006) 25:309–10. 17264434

[10] Domínguez M Moreno I Aizpurua C Toraño A. Early mechanisms of Leishmania infection in human blood. Microbes Infect. (2003) 5:507–13. doi: 10.1016/S1286-4579(03)00071-6 12758280

[11] Brittingham A Morrison CJ McMaster WR McGwire BS Chang K-P Mosser DM. Role of the Leishmania surface protease gp63 in complement fixation, cell adhesion, and resistance to complement-mediated lysis. Parasitol Today. (1995) 11:445–6. doi: 10.1016/0169-4758(95)80054-9 7673725

[12] Pereira-Filho AA Queiroz DC Saab NAA D'Ávila Pessoa GC Koerich LB Pereira MH. Evasion of the complement system by Leishmania through the uptake of C4bBP, a complement regulatory protein, and probably by the action of GP63 on C4b molecules deposited on parasite surface. Acta Trop. (2023) 242:106908. doi: 10.1016/j.actatropica.2023.106908 36963597

[13] Da Silva RP Hall BF Joiner KA Sacks DL. CR1, the C3b receptor, mediates binding of infective Leishmania major metacyclic promastigotes to human macrophages. J Immunol. (1989) 143:617–22. doi: 10.4049/jimmunol.143.2.617 2525590

[14] Jafarzadeh A Nemati M Sharifi I Nair A Shukla D Chauhan P. Leishmania species-dependent functional duality of toll-like receptor 2. IUBMB Life. (2019) 71:1685–700. doi: 10.1002/iub.2129 31329370

[15] Bamigbola IE Ali S. Paradoxical immune response in leishmaniasis: The role of toll-like receptors in disease progression. Parasite Immunol. (2022) 44:e12910. doi: 10.1111/pim.12910 35119120 PMC9285711

[16] van Zandbergen G Klinger M Mueller A Dannenberg S Gebert A Solbach W. Cutting Edge : Neutrophil Granulocyte Serves as a Vector for Leishmania entry into macrophages. J Immunol. (2004) 173:6521–5. doi: 10.4049/jimmunol.173.11.6521 15557140

[17] Sharma S Srivastva S Davis RE Singh SS Kumar R Nylén S. The phenotype of circulating neutrophils during visceral leishmaniasis. Am J Trop Med Hyg. (2017) 97:767–70. doi: 10.4269/ajtmh.16-0722 PMC5590568 28820688

[18] Gueirard P Laplante A Rondeau C Milon G Desjardins M. Trafficking of Leishmania donovani promastigotes in non-lytic compartments in neutrophils enables the subsequent transfer of parasites to macrophages. Cell Microbiol. (2008) 10:100–11. doi: 10.1111/j.1462-5822.2007.01018.x 17651446

[19] Vinet AF Fukuda M Turco SJ Descoteaux A. The Leishmania donovani lipophosphoglycan excludes the vesicular proton-ATPase from phagosomes by impairing the recruitment of Synaptotagmin V. PloS Pathog. (2009) 5:e1000628. doi: 10.1371/journal.ppat.1000628 19834555 PMC2757729

[20] Srivastav S Kar S Chande AG Mukhopadhyaya R Das PK. Leishmania donovani exploits host deubiquitinating enzyme A20, a negative regulator of TLR signaling, to subvert host immune response. J Immunol. (2012) 189:924–34. doi: 10.4049/jimmunol.1102845 22685311

[21] Carneiro MB Vaz LG Afonso LCC Horta MF Vieira LQ. Regulation of macrophage subsets and cytokine production in leishmaniasis. Cytokine. (2021) 147:155309. doi: 10.1016/j.cyto.2020.155309 33334669

[22] Chandrakar P Parmar N Descoteaux A Kar S. Differential induction of SOCS isoforms by leishmania donovani impairs macrophage–T cell cross-Talk and host defense. J Immunol. (2020) 204:596–610. doi: 10.4049/jimmunol.1900412 31882519

[23] Nasseri M Modabber FZ. Generalized infection and lack of delayed hypersensitivity in BALB/c mice infected with Leishmania tropica major. Infect Immun. (1979) 26:611–4. doi: 10.1128/iai.26.2.611-614.1979 PMC414661 546790

[24] Habib S El Andaloussi A Elmasry K Handoussa A Azab M Elsawey A. PDL-1 blockade prevents T cell exhaustion, inhibits autophagy, and promotes clearance of Leishmania donovani. Infect Immun. (2018) 86:e00019–18. doi: 10.1128/IAI.00019-18 29610255 PMC5964517

[25] Hailu A Van Baarle D Knol GJ Berhe N Miedema F Kager PA. T cell subset and cytokine profiles in human visceral leishmaniasis during active and asymptomatic or sub-clinical infection with Leishmania donovani. Clin Immunol. (2005) 117:182–91. doi: 10.1016/j.clim.2005.06.015 16125466

[26] Prajeeth CK Haeberlein S Sebald H Schleicher U Bogdan C. Leishmania-infected macrophages are targets of NK cell-derived cytokines but not of NK cell cytotoxicity. Infect Immun. (2011) 79:2699–708. doi: 10.1128/IAI.00079-11 PMC3191990 21518784

[27] Caldas A Favali C Aquino D Vinhas V van Weyenbergh J Brodskyn C. Balance of IL-10 and interferon-γ plasma levels in human visceral leishmaniasis: Implications in the pathogenesis. BMC Infect Dis. (2005) 5:113. doi: 10.1186/1471-2334-5-113 16364177 PMC1343567

[28] Silva-Barrios S Stäger S. Hypergammaglobulinemia sustains the development of regulatory responses during chronic Leishmania donovani infection in mice. Eur J Immunol. (2019) 49:1082–91. doi: 10.1002/eji.201847917 31001826

[29] Maroof A Beattie L Zubairi S Svensson M Stager S Kaye PM. Posttranscriptional regulation of il10 gene expression allows natural killer cells to express immunoregulatory function. Immunity. (2008) 29:295–305. doi: 10.1016/j.immuni.2008.06.012 18701085 PMC2656759

[30] Liberopoulos E Kei A Apostolou F Elisaf M. Autoimmune manifestations in patients with visceral leishmaniasis. J Microbiol Immunol Infect. (2013) 46:302–5. doi: 10.1016/j.jmii.2012.01.016 22516744

[31] Lindoso JAL Moreira CHV Cunha MA Queiroz IT. Visceral leishmaniasis and HIV coinfection: Current perspectives. HIV/AIDS - Res Palliat. Care. (2018) 10:193–201. doi: 10.2147/HIV.S143929 PMC6197215 30410407

[32] Diro E Lynen L Ritmeijer K Boelaert M Hailu A van Griensven J. Visceral leishmaniasis and HIV coinfection in east africa. PloS Negl Trop Dis. (2014) 8:e2869. doi: 10.1371/journal.pntd.0002869 24968313 PMC4072530

[33] Henn GAL Ramos Júnior AN Colares JKB Mendes LP Silveira JGC Lima AAF. Is Visceral Leishmaniasis the same in HIV-coinfected adults? Braz J Infect Dis. (2018) 22:92–8. doi: 10.1016/j.bjid.2018.03.001 PMC9428234 29601790

[34] Lima IP Müller MC Holanda TA Harhay M Costa CHN Costa DL. Human immunodeficiency virus/Leishmania infantum in the first foci of urban American visceral leishmaniasis: clinical presentation from 1994 to 2010. Rev Soc Bras Med Trop. (2013) 46:156–60. doi: 10.1590/0037-8682-0033-2012 23666663

[35] de O. Santos G de Jesus NPS Cerqueira-Braz JV Santos VS Lemos LMDde. Prevalence of HIV and associated factors among visceral leishmaniasis cases in an endemic area of Northeast Brazil. Rev Soc Bras Med Trop. (2019) 52:e20180257. doi: 10.1590/0037-8682-0257-2018 30892399

[36] Leite de Sousa-Gomes M Romero GAS Werneck GL. Visceral leishmaniasis and HIV/AIDS in Brazil: Are we aware enough? PloS Negl Trop Dis. (2017) 11:e0005772. doi: 10.1371/journal.pntd.0005772 28945816 PMC5612457

[37] Coutinho JVSC Santos FSD Ribeiro RDSP Oliveira IBB Dantas VB Santos ABFS. Visceral leishmaniasis and leishmaniasis-HIV coinfection: comparative study. Rev Soc Bras Med Trop. (2017) 50:670–4. doi: 10.1590/0037-8682-0193-2017 29160515

[38] Costa LDLN Lima US Rodrigues V Lima MIS Silva LA Ithamar J. Factors associated with relapse and hospital death in patients coinfected with visceral leishmaniasis and HIV: a longitudinal study. BMC Infect Dis. (2023) 23:141. doi: 10.1186/s12879-023-08009-1 36882732 PMC9993705

[39] Silva-Freitas ML Cota GF Machado-de-Assis TS Giacoia-Gripp C Rabello A Da-Cruz AM. Immune activation and bacterial translocation: A link between impaired immune recovery and frequent visceral leishmaniasis relapses in HIV-infected patients. PloS One. (2016) 11:e0167512. doi: 10.1371/journal.pone.0167512 27907136 PMC5132299

[40] Casado JL Abad-Fernández M Moreno S Pérez-Elías MJ Moreno A Bernardino JI. Visceral leishmaniasis as an independent cause of high immune activation, T-cell senescence, and lack of immune recovery in virologically suppressed HIV-1-coinfected patients. HIV Med. (2015) 16:240–8. doi: 10.1111/hiv.12206 25604328

[41] Vallejo A Abad-Fernández M Moreno S Moreno A Pérez-Elías MJ Dronda F. High levels of CD4^+^ CTLA-4^+^ Treg cells and CCR5 density in HIV-1-infected patients with visceral leishmaniasis. Eur J Clin Microbiol Infect Dis Off Publ. Eur Soc Clin Microbiol. (2015) 34:267–75. doi: 10.1007/s10096-014-2229-1 25142804

[42] van Griensven J Carrillo E López-Vélez R Lynen L Moreno J. Leishmaniasis in immunosuppressed individuals. Clin Microbiol Infect Off Publ. Eur Soc Clin Microbiol Infect Dis. (2014) 20:286–99. doi: 10.1111/1469-0691.12556 24450618

[43] Clemente WT Rabello A Faria LC Peruhype-Magalhães V Gomes LI da Silva TA. High prevalence of asymptomatic Leishmania sinfection among liver transplant recipients and donors from an endemic area of Brazil. Am J Transplant. Off J Am Soc Transplant. Am Soc Transpl. Surg. (2014) 14:96–101. doi: 10.1111/ajt.12521 24369026

[44] Clemente W Vidal E Girão E Ramos AS Govedic F Merino E. Risk factors, clinical features and outcomes of visceral leishmaniasis in solid-organ transplant recipients: a retrospective multicenter case-control study. Clin Microbiol Infect Off Publ. Eur Soc Clin Microbiol Infect Dis. (2015) 21:89–95. doi: 10.1016/j.cmi.2014.09.002 25636932

[45] Finocchi A Palma P Di Matteo G Chiriaco M Lancella L Simonetti A. Visceral leishmaniasis revealing chronic granulomatous disease in a child. Int J Immunopathol Pharmacol. (2008) 21(3):739–43. doi: 10.1177/039463200802100330 18831944

[46] Al Zayed MSZ. Case study visceral leishmaniasis and chronic granulomatous disease in an infant. Int J Curr Microbiol Appl Sci. (2015) 4:88–91.

[47] Carvalho DG Vasconcelos DM Santos ACR Lindoso JAL. Visceral leishmaniasis revealing undiagnosed inborn errors of immunity. Rev da Sociedade Bras Medicina Trop. (2023) 56:e03222023. doi: 10.1590/0037-8682-0322-2023 PMC10637728 37970879

[48] Diro E Blesson S Edwards T Ritmeijer K Fikre H Admassu H. A randomized trial of AmBisome monotherapy and AmBisome and miltefosine combination to treat visceral leishmaniasis in HIV co-infected patients in Ethiopia. PloS Negl Trop Dis. (2019) 13:e0006988. doi: 10.1371/journal.pntd.0006988 30653490 PMC6336227

[49] Diro E Edwards T Ritmeijer K Fikre H Abongomera C Kibret A. Long term outcomes and prognostics of visceral leishmaniasis in HIV infected patients with use of pentamidine as secondary prophylaxis based on CD4 level: a prospective cohort study in Ethiopia. PloS Negl Trop Dis. (2019) 13:e0007132. doi: 10.1371/journal.pntd.0007132 30789910 PMC6400407

[50] López-Véle R Videla S Márquez M Boix V Jiménez-Mejías ME Górgolas M. Amphotericin B lipid complex versus no treatment in the secondary prophylaxis of visceral leishmaniasis in HIV-infected patients. J Antimicrob Chemother. (2004) 53:540–3. doi: 10.1093/jac/dkh084 14739148

[51] Bin Mannan S Elhadad H Loc TTH Sadik M Mohamed MYF Nam NH. Prevalence and associated factors of asymptomatic leishmaniasis: a systematic review and meta-analysis, Parasitol. Int. (2020) 81:102229. doi: 10.1016/j.parint.2020.102229 33144197

[52] Ortalli M De Pascali AM Longo S Pascarelli N Porcellini A Ruggeri D. Asymptomatic Leishmania infantum infection in blood donors living in an endemic area, northeastern Italy. J Infect. (2020) 80:116–20. doi: 10.1016/j.jinf.2019.09.019 31585188

[53] Chakravarty J Hasker E Kansal S Singh OP Malaviya P Singh AK. Determinants for progression from asymptomatic infection to symptomatic visceral leishmaniasis: A cohort study. PloS Negl Trop Dis. (2018) 13:e0007216. doi: 10.1371/journal.pntd.0007216 PMC6453476 30917114

[54] Da Cunha GMR Carneiro M Pascoal-Xavier MA da Rocha ICM Magalhães FDC Martins-Filho OA. Prospection of immunological biomarkers for characterization and monitoring of asymptomatic Leishmania (Leishmania) infantum infection. Parasitology. (2020) 147:1124–32. doi: 10.1017/S0031182020000852 PMC10317783 32460936

[55] Redhu NS Dey A Balooni V Singh S. Use of immunoglobulin G avidity to determine the course of disease in visceral and post-kala-azar dermal leishmaniasis patients. Clin Vaccine Immunol. (2006) 13:969–71. doi: 10.1128/CVI.00149-06 PMC1539110 16894000

[56] Tiburcio MGS Anversa L Kanunfre KA Ferreira AW Rodrigues V De Silva LA. Anti-leishmania infantum IgG antibody avidity in visceral leishmaniasis. Clin Vaccine Immunol. (2013) 20:1697–702. doi: 10.1128/CVI.00367-13 PMC3837788 24006136

[57] Takele Y Mulaw T Adem E Shaw CJ Franssen SU Womersley R. Immunological factors, but not clinical features, predict visceral leishmaniasis relapse in patients co-infected with HIV. Cell Rep Med. (2022) 3:100487. doi: 10.1016/j.xcrm.2021.100487 35106507 PMC8784791

[58] LeishGEN Consortium Wellcome Trust Case Control Consortium 2 Fakiola M Strange A Cordell HJ Miller EN. Common variants in the HLA-DRB1-HLA-DQA1 HLA class II region are associated with susceptibility to visceral leishmaniasis. Nat Genet. (2013) 45:208–13. doi: 10.1038/ng.2518 PMC3664012 23291585

[59] de Vrij N Meysman P Gielis S Adriaensen W Laukens K Cuypers B. Hla-drb1 alleles associated with lower leishmaniasis susceptibility share common amino acid polymorphisms and epitope binding repertoires. Vaccines. (2021) 9:1–17. doi: 10.3390/vaccines9030270 PMC8002611 33803005

[60] Mahajan R Owen SI Kumar S Pandey K Kazmi S Kumar V. Prevalence and determinants of asymptomatic Leishmania infection in HIV-infected individuals living within visceral leishmaniasis endemic areas of Bihar, India. PloS Negl Trop Dis. (2022) 16:e0010718. doi: 10.1371/JOURNAL.PNTD.0010718 36040931 PMC9467307

[61] Guedes DL Silva EDD Castro MCAB Júnior WLB Ibarra-Meneses AV Tsoumanis A. Comparison of serum cytokine levels in symptomatic and asymptomatic HIV-Leishmania coinfected individuals from a Brazilian visceral leishmaniasis endemic area. PloS Negl Trop Dis. (2022) 16:e0010542. doi: 10.1371/JOURNAL.PNTD.0010542 35714136 PMC9246190

[62] Giorgio S Gallo-Francisco PH Roque GAS Flóro e Silva M. Granulomas in parasitic diseases: the good and the bad. Parasitol Res. (2020) 119:3165–80. doi: 10.1007/s00436-020-06841-x 32789534

[63] Pampiglione S Manson-Bahr PEC Giungi F Giunti G Parenti A Trotti GC. Studies on mediterranean leishmaniasis: 2. asymptomatic cases of visceral leishmaniasis. Trans R Soc Trop Med Hyg. (1974) 68:447–53. doi: 10.1016/0035-9203(74)90067-4 4460309

[64] Singh SS Chauhan SB Ng SS Corvino D de Labastida Rivera F Engel JA. Increased amphiregulin expression by CD4+ T cells from individuals with asymptomatic Leishmania donovani infection. Clin Transl Immunol. (2022) 11:e1396. doi: 10.1002/cti2.1396 PMC9136704 35663920

[65] Amprey JL Im JS Turco SJ Murray HW Illarionov PA Besra GS. A subset of liver NK T cells is activated during Leishmania donovani infection by CD1d-bound lipophosphoglycan. J Exp Med. (2004) 200:895–904. doi: 10.1084/jem.20040704 15466622 PMC2213292

[66] Venuprasad K Banerjee PP Chattopadhyay S Sharma S Pal S Parab PB. Human neutrophil-Expressed CD28 interacts with macrophage B7 to induce phosphatidylinositol 3-Kinase-Dependent IFN-γ Secretion and restriction of leishmania growth. J Immunol. (2002) 169:920–8. doi: 10.4049/jimmunol.169.2.920 12097397

[67] Alizadeh Z Omidnia P Altalbawy FMA Gabr GA Obaid RF Rostami N. Unraveling the role of natural killer cells in leishmaniasis, Int. Immunopharmacol. (2023) 114:109596. doi: 10.1016/j.intimp.2022.109596 36700775

[68] Pitta MGR Romano A Cabantous S Henri S Hammad A Kouriba B. IL-17 and IL-22 are associated with protection against human kala azar caused by Leishmania donovani. J Clin Invest. (2009) 119:2379–87. doi: 10.1172/JCI38813 PMC2719936 19620772

[69] Dirkx L Hendrickx S Merlot M Bulté D Starick M Elst J. Long-term hematopoietic stem cells as a parasite niche during treatment failure in visceral leishmaniasis. Commun Biol. (2022) 5:626. doi: 10.1038/s42003-022-03591-7 35752645 PMC9233693

[70] Mandell MA Beverley SM. Continual renewal and replication of persistent Leishmania major parasites in concomitantly immune hosts. Proc Natl Acad Sci USA. (2017) 114:E801–10. doi: 10.1073/pnas.1619265114 PMC5293024 28096392

[71] Mandell MA Beverley SM. Concomitant immunity induced by persistent leishmania major does not preclude secondary re-infection: implications for genetic exchange, diversity and vaccination. PloS Negl Trop Dis. (2016) 10:1–14. doi: 10.1371/journal.pntd.0004811 PMC4924822 27352043

[72] Saunders EC McConville MJ. Immunometabolism of leishmania granulomas. Immunol Cell Biol. (2020) 98:832–44. doi: 10.1111/imcb.12394 32780446

[73] Terrazas C Varikuti S Oghumu S Steinkamp HM Ardic N Kimble J. Ly6Chi inflammatory monocytes promote susceptibility to Leishmania donovani infection. Sci Rep. (2017) 7:14693. doi: 10.1038/s41598-017-14935-3 29089636 PMC5665970

[74] Osorio EY Zhao W Espitia C Saldarriaga O Hawel L Byus CV. Progressive visceral leishmaniasis is driven by dominant parasite-induced STAT6 activation and STAT6-dependent host arginase 1 expression. PloS Pathog. (2012) 8:e1002417. doi: 10.1371/journal.ppat.1002417 22275864 PMC3261917

[75] Pessenda G da Silva JS. Arginase and its mechanisms in Leishmania persistence. Parasite Immunol. (2020) 42:1–12. doi: 10.1111/pim.12722 32294247

[76] Aronson N Herwaldt BL Libman M Pearson R Lopez-Velez R Weina P. Diagnosis and treatment of leishmaniasis: clinical practice guidelines by the infectious diseases society of america (IDSA) and the american society of tropical medicine and hygiene (ASTMH). Clin Infect Dis. (2016) 63:e202–64. doi: 10.1093/cid/ciw670 27941151

[77] George JT Sadiq M Sigamani E Mathuram AJ. Visceral leishmaniasis with haemophagocytic lymphohistiocytosis. BMJ Case Rep. (2019) 12:e226361. doi: 10.1136/bcr-2018-226361 PMC6381973 30765439

[78] Badiola J Muñoz-Medina L Callejas JL Delgado-García A Jurado M Hernández-Quero J. Hemophagocytic lymphohistiocytosis associated with Leishmania: A hidden passenger in endemic areas. Enfermedades Infecc. y Microbiol Clin. (2021) 39:188–91. doi: 10.1016/j.eimc.2020.04.012 32473845

[79] Jordan MB. Hemophagocytic lymphohistiocytosis: A disorder of T cell activation, immune regulation, and distinctive immunopathology. Immunol Rev. (2023) 322:339–50. doi: 10.1111/imr.13298 38100247

[80] Tapisiz A Belet N Ciftçi E Ince E Dogru U. Hemophagocytic lymphohistiocytosis associated with visceral leishmaniasis. J Trop Pediatr. (2007) 53:359–61. doi: 10.1093/tropej/fmm024 17626064

[81] Rajagopala S Dutta U Chandra KSP Bhatia P Varma N Kochhar R. Visceral leishmaniasis associated hemophagocytic lymphohistiocytosis–case report and systematic review. J Infect. (2008) 56:381–8. doi: 10.1016/j.jinf.2008.02.013 18405976

[82] Horrillo L Castro A Matía B Molina L García-Martínez J Jaqueti J. Clinical aspects of visceral leishmaniasis caused by L. infantum in adults. Ten years of experience of the largest outbreak in Europe: what have we learned? Parasitol Vectors. (2019) 12:359. doi: 10.1186/s13071-019-3628-z PMC6657057 31340851

[83] Chandra H Chandra S Kaushik RM. Visceral leishmaniasis with associated common, uncommon, and atypical morphological features on bone marrow aspirate cytology in nonendemic region. J Trop Med. (2013) 2013:861032. doi: 10.1155/2013/861032 24089618 PMC3782059

[84] Chaturvedi V Marsh RA Zoref-Lorenz A Owsley E Chaturvedi V Nguyen TC. T-cell activation profiles distinguish hemophagocytic lymphohistiocytosis and early sepsis. Blood. (2021) 137:2337–46. doi: 10.1182/blood.2020009499 PMC8085480 33512385

[85] De Matteis A Colucci M Rossi MN Caiello I Merli P Tumino N. Expansion of CD4dimCD8+ T cells characterizes macrophage activation syndrome and other secondary HLH. Blood. (2022) 140:262–73. doi: 10.1182/blood.2021013549 35500103

[86] Nguyen TH Kumar D Prince C Martini D Grunwell JR Lawrence T. Frequency of HLA-DR(+)CD38(hi) T cells identifies and quantifies T-cell activation in hemophagocytic lymphohistiocytosis, hyperinflammation, and immune regulatory disorders. J Allergy Clin Immunol. (2024) 153:309–19. doi: 10.1016/j.jaci.2023.07.008 PMC10823038 37517575

[87] Mastrolia MV Boscia S Galli L Lodi L Pisano L Maccora I. CD38high/HLA-DR+ CD8+ T cells as potential biomarker of hemophagocytic lymphohistiocytosis secondary to visceral Leishmania infection. Eur J Pediatr. (2023) 182:1429–32. doi: 10.1007/s00431-022-04789-x 36631689

[88] Mottaghipisheh H Kalantar K Amanati A Shokripour M Shahriari M Zekavat OR. Comparison of the clinical features and outcome of children with hemophagocytic lymphohistiocytosis (HLH) secondary to visceral leishmaniasis and primary HLH: a single-center study. BMC Infect Dis. (2021) 21:732. doi: 10.1186/s12879-021-06408-w 34340686 PMC8330039

[89] Bode SFN Bogdan C Beutel K Behnisch W Greiner J Henning S. Hemophagocytic lymphohistiocytosis in imported pediatric visceral leishmaniasis in a nonendemic area. J Pediatr. (2014) 165:147–153.e1. doi: 10.1016/j.jpeds.2014.03.047 24797953

[90] Chellapandian D Das R Zelley K Wiener SJ Zhao H Teachey DT. Treatment of Epstein Barr virus-induced haemophagocytic lymphohistiocytosis with rituximab-containing chemo-immunotherapeutic regimens. Br J Haematol. (2013) 162:376–82. doi: 10.1111/bjh.12386 PMC3776423 23692048

[91] Cançado GGL Freitas GG Faria FHF de Macedo AV Nobre V. Hemophagocytic lymphohistiocytosis associated with visceral leishmaniasis in late adulthood. Am J Trop Med Hyg. (2013) 88:575–7. doi: 10.4269/ajtmh.12-0563 PMC3592544 23324220

[92] Matnani R Ganapathi KA. Hemophagocytic lymphohistiocytosis associated with visceral leishmaniasis. Blood. (2016) 127:513. doi: 10.1182/blood-2015-10-678862 27218126

[93] Scalzone M Ruggiero A Mastrangelo S Trombatore G Ridola V Maurizi P. Hemophagocytic lymphohistiocytosis and visceral leishmaniasis in children: case report and systematic review of literature. J Infect Dev Ctries. (2016) 10:103–8. doi: 10.3855/jidc.6385 26829545

[94] López Marcos M Ruiz Sáez B Vílchez Pérez JS Moreno Pérez D Carazo Gallego B Falcón Neyra L. Distinct laboratory and clinical features of secondary hemophagocytic lymphohistiocytosis in pediatric visceral leishmaniasis: A retrospective analysis of 127 children in andalusia, Spain (2004-2019). Pediatr Infect Dis J. (2021) 40:525–30. doi: 10.1097/INF.0000000000003086 33538542

[95] La Rosée P Horne A Hines M von Bahr Greenwood T Machowicz R Berliner N. Recommendations for the management of hemophagocytic lymphohistiocytosis in adults. Blood. (2019) 133:2465–77. doi: 10.1182/blood.2018894618 30992265

[96] George JT Sadiq M Sigamani E Mathuram AJ. Visceral leishmaniasis with haemophagocytic lymphohistiocytosis. BMJ Case Rep. (2019) 12:2018–20. doi: 10.1136/bcr-2018-226361 PMC6381973 30765439

[97] Daher EF Lima LL Vieira AP Nascimento LS Soares DS Abreu KL. Hemophagocytic syndrome in children with visceral leishmaniasis. Pediatr Infect Dis J. (2015) 34:1311–4. doi: 10.1097/INF.0000000000000916 26780020

[98] Blázquez-Gamero D Domínguez-Pinilla N Chicharro C Negreira S Galán P Pérez-Gorricho B Calvo C. Hemophagocytic lymphohistiocytosis in children with visceral leishmaniasis. Pediatr Infect Dis J. (2015) 34:667–9. doi: 10.1097/INF.0000000000000685 25970110

[99] Brisse E Wouters CH Matthys P. Advances in the pathogenesis of primary and secondary haemophagocytic lymphohistiocytosis: differences and similarities. Br J Haematol. (2016) 174:203–17. doi: 10.1111/bjh.14147 27264204

[100] Bryceson YT Pende D Maul-Pavicic A Gilmour KC Ufheil H Vraetz T. A prospective evaluation of degranulation assays in the rapid diagnosis of familial hemophagocytic syndromes. Blood. (2012) 119:2754–63. doi: 10.1182/blood-2011-08-374199 22294731

[101] Grom AA. Natural killer cell dysfunction: A common pathway in systemic-onset juvenile rheumatoid arthritis, macrophage activation syndrome, and hemophagocytic lymphohistiocytosis? Arthritis Rheumatol. (2004) 50:689–98. doi: 10.1002/art.20198 15022306

[102] Al-Samkari H Berliner N. Hemophagocytic lymphohistiocytosis. Annu Rev Pathol. (2018) 13:27–49. doi: 10.1146/annurev-pathol-020117-043625 28934563

[103] Canna SW Marsh RA. Pediatric hemophagocytic lymphohistiocytosis. Blood. (2020) 135:1332–43. doi: 10.1182/blood.2019000936 PMC8212354 32107531

[104] Shi Q Huang M Li X Zheng X Wang F Zou Y. Clinical and laboratory characteristics of hemophagocytic lymphohistiocytosis induced by Leishmania infantum infection. PloS Negl Trop Dis. (2021) 15:e0009944. doi: 10.1371/journal.pntd.0009944 34735436 PMC8594843

[105] Machelart I Lapoirie J Viallard JF Mirabel M Lazaro E Greib C. Visceral leishmaniasis with hemophagocytic lymphohistiocytosis in an immunocompetent adult. Med Mal. Infect. (2019) 49:548–50. doi: 10.1016/j.medmal.2019.07.006 31326300

[106] Gera A Misra A Tiwari A Singh A Mehndiratta S. A hungry Histiocyte, altered immunity and myriad of problems: Diagnostic challenges for Pediatric HLH. Int J Lab Hematol. (2021) 43:1443–50. doi: 10.1111/ijlh.13626 34118134

[107] Mantadakis E Alexiadou S Totikidis G Grapsa A Chatzimichael A. A brief report and mini-review of visceral leishmaniasis-associated hemophagocytic lymphohistiocytosis in children. J Pediatr Hematol Oncol. (2021) 43:e223–e226. doi: 10.1097/MPH.0000000000001747 32049769

[108] Brum NFF Coelho JS Carvalho LS Vieira MNO Bentes AA Carellos EVM. Hemophagocytic lymphohistiocytosis and visceral leishmaniasis in children: a series of cases and literature review. Rev Paul. Pediatr. (2021) 40:e2020269. doi: 10.1590/1984-0462/2022/40/2020269 34495274 PMC8432228

[109] Martín A Marques L Soler-Palacín P Caragol I Hernandez M Figueras C. Visceral leishmaniasis associated hemophagocytic syndrome in patients with chronic granulomatous disease. Pediatr Infect Dis J. (2009) 28:753–4. doi: 10.1097/INF.0b013e31819c6f3a 19633526

[110] Zijlstra EE Kumar A Sharma A Rijal S Mondal D Routray S. Report of the fifth post-kala-azar dermal leishmaniasis consortium meeting, colombo, Sri Lanka, 14-16 may 2018. Parasites Vectors. (2020) 13:1–14. doi: 10.1186/s13071-020-04011-7 32228668 PMC7106569

[111] Dixit KK Singh R Salotra P. Advancement in molecular diagnosis of post kala-azar dermal leishmaniasis. Indian J Dermatol. (2020) 65:465–72. doi: 10.4103/ijd.IJD_311_19 PMC7810074 33487701

[112] Ismail A Khalil EA Musa AM El Hassan IM Ibrahim ME Theander TG. The pathogenesis of post kala-azar dermal leishmaniasis from the field to the molecule: does ultraviolet light (UVB) radiation play a role? Med Hypotheses. (2006) 66:993–9. doi: 10.1016/j.mehy.2005.03.035 16386855

[113] Sengupta R Mukherjee S Moulik S Mitra S Chaudhuri SJ Das NK. *In-situ* immune profile of polymorphic vs. macular Indian Post Kala-azar dermal leishmaniasis. Int J Parasitol Drugs Drug Resist. (2019) 11:166–76. doi: 10.1016/j.ijpddr.2019.08.005 PMC6904817 31542359

[114] Singh OP Hasker E Boelaert M Sacks D Sundar S. Xenodiagnosis to address key questions in visceral leishmaniasis control and elimination. PloS Negl Trop Dis. (2020) 14:e0008363. doi: 10.1371/journal.pntd.0008363 32790716 PMC7425851

[115] Mondal D Bern C Ghosh D Rashid M Molina R Chowdhury R. Quantifying the infectiousness of post-kala-azar dermal leishmaniasis toward sand flies. Clin Infect Dis an Off Publ. Infect Dis Soc Am. (2019) 69:251–8. doi: 10.1093/cid/ciy891 PMC6603265 30357373

[116] Molina R Ghosh D Carrillo E Monnerat S Bern C Mondal D. Infectivity of post-kala-azar dermal leishmaniasis patients to sand flies: revisiting a proof of concept in the context of the kala-azar elimination program in the Indian subcontinent. Clin Infect Dis. (2017) 65:150–3. doi: 10.1093/cid/cix245 PMC5848257 28520851

[117] Ramesh V Kaushal H Mishra AK Singh R Salotra P. Clinico-epidemiological analysis of Post kala-azar dermal leishmaniasis (PKDL) cases in India over last two decades: a hospital based retrospective study. BMC Public Health. (2015) 15:1092. doi: 10.1186/s12889-015-2424-8 26503551 PMC4621871

[118] Gedda MR Singh B Kumar D Singh AK Madhukar P Upadhyay S. Post kala-azar dermal leishmaniasis: A threat to elimination program. PloS Negl Trop Dis. (2020) 14:e0008221. doi: 10.1371/journal.pntd.0008221 32614818 PMC7332242

[119] Volpedo G Pacheco-Fernandez T Holcomb EA Cipriano N Cox B Satoskar AR. Mechanisms of immunopathogenesis in cutaneous leishmaniasis and post kala-azar dermal leishmaniasis (PKDL). Front Cell Infect Microbiol. (2021) 11:685296. doi: 10.3389/fcimb.2021.685296 34169006 PMC8217655

[120] Kumar R Singh N Gautam S Singh OP Gidwani K Rai M. Leishmania specific CD4 T cells release IFNγ that limits parasite replication in patients with visceral leishmaniasis. PloS Negl Trop Dis. (2014) 8:e3198. doi: 10.1371/journal.pntd.0003198 25275531 PMC4183461

[121] Mukhopadhyay D Mukherjee S Roy S Dalton JE Kundu S Sarkar A. M2 polarization of monocytes-macrophages is a hallmark of Indian post kala-azar dermal leishmaniasis. PloS Negl Trop Dis. (2015) 9:e0004145. doi: 10.1371/journal.pntd.0004145 26496711 PMC4619837

[122] Mukherjee S Sengupta R Mukhopadhyay D Braun C Mitra S Roy S. Impaired activation of lesional CD8(+) T-cells is associated with enhanced expression of Programmed Death-1 in Indian Post Kala-azar Dermal Leishmaniasis. Sci Rep. (2019) 9:13997. doi: 10.1038/s41598-018-37144-y 30679687 PMC6345993

[123] Ismail A Gadir AFA Theander TG Kharazmi A El Hassan AM. Pathology of post-kala-azar dermal leishmaniasis: a light microscopical, immunohistochemical, and ultrastructural study of skin lesions and draining lymph nodes. J Cutan. Pathol. (2006) 33:778–87. doi: 10.1111/j.1600-0560.2006.00531.x 17177937

[124] Gasim S Elhassan AM Khalil EA Ismail A Kadaru AM Kharazmi A. High levels of plasma IL-10 and expression of IL-10 by keratinocytes during visceral leishmaniasis predict subsequent development of post-kala-azar dermal leishmaniasis. Clin Exp Immunol. (1998) 111:64–9. doi: 10.1046/j.1365-2249.1998.00468.x PMC1904865 9472662

[125] Ritmeijer K Veeken H Melaku Y Leal G Amsalu R Seaman J. Ethiopian visceral leishmaniasis: generic and proprietary sodium stibogluconate are equivalent; HIV co-infected patients have a poor outcome. Trans R Soc Trop Med Hyg. (2001) 95:668–72. doi: 10.1016/s0035-9203(01)90110-5 11816442

[126] Zijlstra EE. PKDL and other dermal lesions in HIV co-infected patients with Leishmaniasis: review of clinical presentation in relation to immune responses. PloS Negl Trop Dis. (2014) 8:e3258. doi: 10.1371/journal.pntd.0003258 25412435 PMC4238984

[127] Salih MAM Fakiola M Abdelraheem MH Younis BM Musa AM ElHassan AM. Insights into the possible role of IFNG and IFNGR1 in Kala-azar and Post Kala-azar Dermal Leishmaniasis in Sudanese patients. BMC Infect Dis. (2014) 14:662. doi: 10.1186/s12879-014-0662-5 25466928 PMC4265480

[128] Dey A Singh S. Genetic heterogeneity among visceral and post-Kala-Azar dermal leishmaniasis strains from eastern India. Infect Genet EJ. Mol Epidemiol. EGenet. Infect Dis. (2007) 7:219–22. doi: 10.1016/j.meegid.2006.09.001 17027344

[129] WHO. “Post-kala-azar dermal leishmaniasis: a manual for case management and control,” Rep. a WHO consultative Meet, Kolkata, India, July, 2–3, 2012, WHO Document Production Services, Geneva, Switzerland.

[130] Riebenbauer K Czerny S Egg M Urban N Kinaciyan T Hampel A. The changing epidemiology of human leishmaniasis in the non-endemic country of Austria between 2000 to 2021, including a congenital case. PloS Negl Trop Dis. (2024) 18:e0011875. doi: 10.1371/journal.pntd.0011875 38198499 PMC10805284

