# Supplementary material for: Immune response to viscerotropic Leishmania: a comprehensive review
Source: Front Immunol. 2024 Sep 18;15:1402539. doi: 10.3389/fimmu.2024.1402539 (PMC11445144; doi:10.3389/fimmu.2024.1402539)
Supplement: Supplementary file 1 [file Table1.docx]

**Supplementary material**

| **References** | **Type of study** | **Population** | **Main findings** |
| --- | --- | --- | --- |
| Dominguez et al, 2003 [10] | Preclinical study | In vitro models | Promastigote lysis by complement parallels C3 deposition kinetics, and ~90% of promastigotes are killed after 2.5 min. |
| Britthingam et al,1995 [11] | Preclinical studies | In vitro models | Organisms expressing surface protein gp63 can exploit the opsonic properties of complement while avoiding its lytic effects. |
| Pereira-Filho et al, 2023 [12] | Preclinical study | In vitro models | Several *Leishmania* strains including *L.infantum* binds host’s serum factors such as factor H and C4bBP to inactivate C4b molecules |
| Da Silva et al, 1989 [13] | Preclinical study | In vitro models | Infective *Leishmania* relies on serum complement proteins for attachment to human macrophages through complement receptor 1 (CR1) without triggering the respiratory burst. |
| Jafarzadeh et al, 2019 [14] | Critical review |  | *L.major* and *L*.*donovani* infections trigger Toll like receptor (TLR) 2-related host-protective and non-protective immune responses. *L*.*mexicana* and *L*.*infantum* infections are reported to elicit TLR2-mediated host-protective responses. |
| van Zandbergen et al, 2004 [16] | Preclinical study | In vitro models | *L.major* is able to silently invade macrophages who phagocytose infected neutrophils. This is called the Trojan Horse mechanism. |
| Sharma et al, 2017 [17] | Cohort study | 37 VL patients from Bihar, India (> 6 years of age; HIV-negative) and 16 controls (healthy patients’ household members) | VL patients’ neutrophils expressed decreased levels of neutrophil chemoattractant CXCL8 (C-X-C motif chemokine ligand 8), increased Interleukin-10, and elevated transcripts for arginase-1 (which suppresses T cell responses) when compared to healthy controls. |
| Gueirard et al, 2008 [18] | Preclinical study | In vitro models | After phagocytosis by neutrophils, *L.donovani* is either degraded within lytic compartments or evades the cytolytic mechanism by hiding in non-lytic compartments, preventing lysosomal fusion. This evasion, facilitated by promastigote surface lipophosphoglycan (LPG), enables *Leishmania* to persist within neutrophils and eventually enter macrophages. |
| Vinet et al, 2009 [19] | Preclinical study | In vitro models | *L.donovani* promastigotes exploit surface lipophosphoglycan to exclude from the forming phagosome the vesicular proton-ATPase resulting in failure to acidify. |
| Srivastas et al, 2012 [20] | Preclinical study | In vitro and mouse models | *L.donovani* exploits host deubiquitinating enzyme to inhibit the Toll Like Receptor 2-mediated proinflammatory gene expression, ultimately leading to suppression of pro-inflammatory cytokines (Interleukin-12 and Tumor Necrosis Factor-α) production. |
| Carneiro et al, 2021 [21] | Narrative review |  | *Leishmania* interaction with host’s macrophages with a focus on escape mechanisms. |
| Chandrakar et al, 2020 [22] | Preclinical study | In vitro and mouse models | Bone marrow derived macrophages and CD4+ T cells infected with *L.donovani* express suppressor of cytokine signaling. The silencing of these suppressors reduced liver and spleen parasite burden in susceptible mouse models (BALB/c mice) (77.1 and 74.8% reduction of liver and spleen parasite burden, respectively). |
| Nasseri et al, 1979 [23] | Preclinical study | Mouse models | Susceptible mouse models exhibited disseminated infection following subcutaneous injection of *L*.*major*, resulting in 100% mortality. In contrast, six other different murine strains developed cutaneous infections that spontaneously resolved. |
| Habiba et al, 2018 [24] | Preclinical study | Mouse models | BALB/c mice infected with *L.donovani* presented on flow cytometry a lower frequency of Interferon-γ, Tumor Necrosis Factor-α CD4+ T helper 1 cells and Interleukin-17 T Helper 17 cells at 21 days post infection, compared to controls and infected mice at 7 days post infection. CD4+ T cells showed a higher level of cytotoxic T-lymphocyte–associated antigen 4 (CTLA-4) and programmed death 1 (PD1) expression at 21 days post infection compared to controls and infected mice at 7 days post infection. The percentage of programmed death ligand 1 (PDL-1) -expressing macrophages in the spleen and bone marrow was higher than that in the controls. Treatment with anti-PDL-1 antibody resulted in restoration of pro-inflammatory cytokines profile and clearance of the parasite from the spleen and bone marrow. |
| Hailu et al, 2005 [25] | Retrospective cohort study | 33 VL patients, 9 treated VL patients, 14 individuals with sub-clinical infection, 34 individuals with asymptomatic infection, and 19 healthy controls from South-West Ethiopia. | Both CD4+ and CD8+ T cells of VL patients could not produce Interferon-γ (IFN-γ) or Interleukin-4 (IL-4) even after mitogen stimulation, this finding was reversed upon cure. The VL patients presented high levels of IFN-γ and IL-10 and reduced levels of memory T cells (CD3+ CD45RO+). |
| Prajeeth et al, 2011 [26] | Preclinical study | In vitro and mouse models | Macrophagic cytotoxicity by natural killer (NK) was only mediated through soluble products and not via direct lysis. Comparative measurement of IFN-γ in macrophage cultures versus NK cell/macrophage co-cultures identified NK cells as the primary source of IFN-γ. |
| Caldas et al, 2005 [27] | Prospective cohort study | 20 patients with VL before and after treatment were compared to 19 healthy volunteers from an endemic area in Brazil; follow-up lasted 12 months. | Elevated plasma levels of IFN-γ, IL-12, p40 and IL-10 were observed during active disease and decreasing after treatment. The activity of IFN-γ is most probably blunted by the elevated levels of IL-10. |
| Silva-Barrios et al, 2019 [28] | Preclinical study | Mouse models | Aicda -/- mice are unable to produce hypermutated and/or class-switched antibodies; these mice were significantly more resistant to *L.donovani* infection compared to wild type mice. Resistance correlated with higher T helper 1 response, less TNFα, IL-10 and INF-β, despite persistence of hypergammaglobulinemia and high levels of IgM. |
| Maroof et al, 2008 [29] | Preclinical study | Mouse models | BALB/c mice infected with *L.donovani* display NK cells recruitment in hepatic and splenic granulomas, where they secrete IL-10 later on in the course of the infection as a consequence of extensive activation. |
| Liberopoulos et al, 2013 [30] | Prospective cohort study | 16 VL patients in Greece | Elevated titers of serum ANA were reported in 88% of patients. Two patients had elevated titers of anti-ENA antibodies and two others had elevated titers of anti-ENA and anti-Ro antibodies. Elevated titers of serum rheumatoid factor in 63%, whereas C3 and C4 factors of complement were decreased in 13% and 50% of patients, respectively. All laboratory autoimmune manifestations were resolved 3 months after therapy. |
| Lindoso et al, 2018 [31] | Narrative review |  | Atypical presentations have been noted in HIV-infected patients during active VL, alongside changes in immune response that lead to heightened immunosuppression. Serological detection of VL in HIV-infected individuals is poor. Given the elevated mortality rates, a combination of medications along with secondary prophylaxis, in conjunction with highly active antiretroviral therapy, emerges as the optimal approach for managing HIV coinfection. |
| Diro et al, 2014 [32] | Narrative review |  | In East Africa, the confluence of VL and HIV coinfection presents a significant contemporary challenge. Practical application of liposomal amphotericin B alongside miltefosine, followed by secondary prophylaxis and antiretroviral therapy, shows encouraging outcomes. |
| de L. Henn et al, 2018 [33] | cross-sectional study | 81 HIV-VL patients and 365 VL controls in Brazil, median age 38 years | In both groups, classic symptoms were prevalent, but the combination of fever, constitutional symptoms, and splenomegaly was more frequent in the VL group compared to the VL-HIV group (86.7% vs. 75.9%, p = 0.01). Patients in the VL-HIV group reported a median duration of symptoms attributable to the disease of three months (range, 0.4–24), compared to two months in the VL group. The positivity of bone marrow aspirate was higher in the VL-HIV group, while the anti-rK39 test performed better in the VL group. |
| Lima et al, 2013 [34] | Retrospective and prospective observational study | 224 patients with VL-HIV coinfection between 20-40 years of age in South America | The most prevalent symptoms and signs included pallor, fever, asthenia, and hepatosplenomegaly. A relapse occurred in 10.2% of cases, and 16.8% of patients in the cohort died. The main risk factors linked to death were kidney or respiratory failure, somnolence, hemorrhagic manifestations, and systemic inflammation syndrome. |
| de O. Santos et Al, 2019 [35] | Observational cross-sectional study | 917 Brazilian patients with VL reported in Sergipe from 1999 to 2015. | 41 patients (4.5%) of them had VL-HIV co-infection. VL-HIV co-infected patients were more likely to have weight loss, cough, treatment failure or loss to follow-up and death. |
| de Sousa-Gomes et al, 2017 [36] | Retrospective observational study | 35.819 VL cases registered in Brazil since 2001 to 2010; 760 (2.12%) were VL/AIDS coinfected patients, 541 (1.51%) were (VL/HIV coinfected patients and 34,518 (96.37%) non-coinfected patients. | Asthenia, weight loss, cough, additional infections, and hemorrhagic events were prevalent among patients with coinfection, resulting in a mortality rate three times higher than that of non-coinfected individuals. Moreover, the relapse rate was twice as high among those with coinfection (6.3%) compared to those without (3.1%). |
| Coutinho et al, 2017 [37] | Retrospective study | 473 cases of VL reported in Araguaína, Tocantins, Brazil between 2009 and 2014 | 5.5% were coinfected with HIV. A higher proportion of VL cases was seen in children aged 0-10 years, whereas coinfection was more common in those aged 18-50 years. In individuals with VL alone and those with VL/HIV coinfection, fever was reported in 97.76% and 92.3% of cases, respectively, while splenomegaly occurred in 68.7% and 61.5% of cases, respectively. However, a significantly higher percentage of patients in the coinfection group experienced physical weakness and weight loss, whereas a greater percentage of patients in the VL group presented with pallor (43.2%) |
| Costa et al, 2023 [38] | Prospective longitudinal study | 169 patients coinfected with VL and HIV in Brazil | The relapse rate for VL was 41.4%, with a death rate of 11.2%. Splenomegaly and adenomegaly were linked to a higher risk of VL relapse. Patients experiencing relapse had elevated urea and creatinine levels, while those who died had lower red blood cell counts, hemoglobin levels, and platelet counts. The adjusted model revealed that antiretroviral therapy for over six months reduced the risk of VL relapse. |
| Silva-Freitas et al, 2016 [39] | Prospective cohort study | 18 VL-HIV co-infected patients were recruited in Brazil, from February 2011 to March 2013. VL/HIV patients were divided into non-relapsing (NR, n = 6) and relapsing (R, n = 11) groups | All patients were undergoing highly active antiretroviral therapy (HAART) and secondary prophylaxis post-VL treatment. During active VL, both groups exhibited similar immunological parameters, including parasite load. At 6 and 12 months after treatment, the NR group displayed significant increases in CD4+ T cells, reduced lymphocyte activation, and lower levels of soluble CD14 and anti-*Leishmania* IgG3 compared to the R group. Viral load remained low without correlation to activation. The R group may have a diminished ability to regulate immune activation compared to the NR group. |
| Casado et al, 2015 [40] | Cross-sectional study | 55 HIV patients receiving suppressive combination antiretroviral therapy (cART) for at least 1 year: 9 with previous relapsing VL, 20 with an immune discordant response (IDR) to cART (CD4 count<200 cells/μL) and no previous VL, and 26 with a concordant response (CR) to cART (CD4 count>350 cells/μL) without VL. | Patients previously diagnosed with VL showed heightened levels of CD8 T-cell activation, along with elevated markers of inflammation and monocyte activation such as IL-6, LPS, and sCD14, compared to those with initial diagnosis and treatment response (IDR). Additionally, patients with prior VL exhibited increased CD8 T-cell senescence. Notably, the levels of immune activation and inflammation in patients with previous VL were not associated with the timing of VL diagnosis, the frequency of VL relapses, or hepatitis C virus (HCV) coinfection. |
| Vallejo et al, 2015 [41] | Cross-sectional study | 9 VL-HIV patients receiving suppressive cART for at least 1 year, compared to 16 HIV patients with non-immunological response (CD4 count below 250 cells/mm3) and 26 HIV infected patients with immunological response (CD4 count above 500 cells/mm3) without VL | VL-HIV patients had more depleted naïve T cells, elevated CD4 regulatory T cells, increased levels of CD4 Treg CTLA-4+ cells, and higher CCR5 density on T cells, all potentially contributing to persistent and recurrent VL. |
| van Griensven et al, 2015 [42] | Review |  | In immunosuppressed individuals, clinical signs may be unusual and misinterpreted as disease flare-ups. Combining parasitological and serological/molecular methods provides the best diagnosis. Liposomal amphotericin B is the preferred treatment, but relapse rates are high in HIV coinfection. Secondary prophylaxis is recommended for immunocompromised patients. |
| Clemente et al, 2014 [43] | Cross-sectional study | 50 liver recipients and 17 liver donors tested for VL in Brazil | Liver and spleen tissues showed no *Leishmania* spp. amastigotes. Among 67 serum samples, 1.5% were positive by indirect fluorescent antibody test, 17.9% were indeterminate, and the rK39 rapid test was negative. Polymerase Chain Reaction (PCR) detected *Leishmania* DNA in 7.5%, 8.9%, and 5.9% of blood, liver, and spleen samples, respectively, confirming 23.5% of donors and 8% of recipients as positive. Specific PCR confirmed all positive samples. These findings indicate a high prevalence of asymptomatic *L.infantum*, with PCR being the most sensitive screening method. |
| Clemente et al, 2015 [44] | Multicenter case–control study | 25139 solid-organ transplant recipients from January 1995 to June 2012 and January  1995 to December 2011 at the participating transplant centers  in Spain and Brazil | 36 VL cases were identified (0.1%).  In Brazil VL is significantly more common than in Spain, often occurring around 11 months post-transplantation. High-dose prednisone use in the previous 6 months is linked to VL. Diagnosis is often delayed, with many patients presenting with fever, visceromegaly, and pancytopenia. Co-infections are common. Treatment typically involves amphotericin B, with relapses occurring in about a quarter of cases and a crude mortality rate of 2.8%. |
| Finocchi et al, 2008 [45] | Case report | 3-year-old child with VL and chronic granulomatous disease (CGD) | After VL diagnosis, Standard treatment with intravenous liposomal amphotericin B was started. Due to the poor response to treatment, an immunodeficiency disorder was suspected and the nitroblue tetrazolium (NBT) test was performed, showing an impaired neutrophils’ function. IFN-γ was started, with a recovery of the patient. Subsequently, a genetic CGD diagnosis was done. |
| Al Zayed et al, 2015 [46] | Case report | 6-month-old infant with disseminated VL and CGD | The child presented with fever and skin lesion. After a bone marrow aspirate, VL diagnosis was done. He was initially treated with Amphotericin B and then received living related donor bone marrow transplantation. The oxidative burst test was zero, confirming CGD. |
| Carvalho et al, 2023 [47] |  | 2 immunocompromised patients with VL | After VL diagnosis, the first patient was treated with standard L-amphotericin B regimen, but had a poor response. He was investigated for Inborn Error of Immunity (IEI) and GATA2 deficiency was detected.  The second patient presented 2 VL relapses, after the first infection. He was investigated and Griscelli Syndrome was detected |
| Diro et al, 2019 [48] | Randomized clinical trial | 59 VL-HIV Ethiopian patients, 20 were treated with L-amphotericin B alone, 39 were treated with a combination of L-amphotericin B and miltefosine | The combination treatment showed a higher efficacy than the monotherapy. Extended treatment based on initial response achieved a 91% parasite clearance rate, particularly beneficial for relapse patients. Parasitological assessment at the end of treatment was crucial, detecting treatment failures missed by clinical evaluation. Adverse events were mostly mild and did not lead to treatment discontinuation, suggesting a satisfactory safety profile even with prolonged treatment. |
| Diro et al, 2019 [49] | Prospective cohort study | 54 VL-HIV Ethiopian patients monitored for 1 year after VL treatment. | The probability of relapse-free survival at one year was 50%. 22 patients with CD4 ≥200 cells/μL who did not receive pentamidine prophylaxis had a 53% probability, while 29 patients with CD4 <200 cells/μL who started pentamidine had a 46% probability of relapse-free survival. Among those with CD4 ≥200 cells/μL, VL relapse independently increased the risk of subsequent relapse or death. |
| López-Vélez et al, 2004 [50] | Multicenter clinical trial | 17 HIV-infected patients, with at least one previous treated episode of VL and with negative bone marrow aspirate for *Leishmania* parasites prior to the study, were randomized to receive either amphotericin B lipid complex 3 mg/kg/day every 21 days (ABLC) or no treatment (NT). | 50% of patients in the ABLC group remained free of VL after 12 months, compared to 22.2% in the NT group. ABLC was well tolerated, with patients experiencing only mild infusion-related adverse events. No patients in either group discontinued treatment or died during the follow-up period. |

**Table S1.** Immunity and symptomatic visceral leishmaniasis - Summary of the articles cited. **Abbreviations**: VL, Visceral Leishmaniasis.

| **References** | **Type of study** | **Population** | **Main findings** |
| --- | --- | --- | --- |
| Mannan et al, 2021 [51] | Systematic review and meta-analysis | Based on 111 studies | The prevalence of asymptomatic leishmaniasis was 11.2% in the general population and 11.8% in HIV patients. Among individuals with leishmaniasis, 64.9% were asymptomatic. |
| Ortalli et al, 2020 [52] | Cross-sectional study | 240 healthy blood donors in Northeastern Italy | The prevalence of asymptomatic *Leishmania* infection in the blood donor cohort was 12.5%, detected with Western blot serological analysis. While *Leishmania* DNA was detected with real time Polymerase Chain Reaction (PCR) assay in 1.7% of donors. |
| Chakravarty et al. 2018 [53] | Prospective Cohort study | 1606 healthy subjects of > 2 years of age in Bihar, India. 476 of them had recent seroconversion, 1130 were heathy controls. | High direct antiglobulin test (DAT), rK39- enzyme-linked immunoassay (ELISA) antibody titers and quantitative PCR are associated with progression from seroconversion to VL (odds ratios 19.1, 30.3 and 20.9 respectively). Most VL cases arose early (median 5 months) during follow-up. |
| Da Cunha et al, 2020 [54] | Cross-sectional study | 179 asymptomatic children aged 1-10 years old from an endemic region of Brazil divided in three cohorts: asymptomatic, VL or uninfected | Lower avidity rates and Interferon-γ (INF-γ) production associated with higher Interleukin-17A (IL-17A) levels are hallmarks of asymptomatic *L*.*infantum* infections. |
| Redhu et al, 2006 [55] | Cohort study | Serum samples from 50 parasitologically confirmed patients with leishmaniasis were compared to serum samples from 50 healthy volunteers and those of 150 patients with other illnesses including HIV, in New Delhi, India. | The study showed that avidity could be used to determine the duration of leishmaniasis. More than 76% of patients with an illness duration of less than 6 months had avidity of less than 70%, 94% of patients had less than 80% avidity, and all patients with illness of more than 6 months had avidity values higher than 70%. |
| Tiburcio et al, 2013 [56] | Cohort study | Serum samples taken from 10 patients with visceral leishmaniasis, 18 cured patients and 20 asymptomatic subjects with at least one positive *Leishmania* test in an endemic area of Brazil. | The proportion of high-avidity antibodies was higher in all samples from patients with VL. In contrast, low-avidity antibodies predominated in subjects with a history of VL and asymptomatic subjects. The frequency of high-avidity antibodies increased over time in 35% of asymptomatic patients. |
| Takele et al, 2022 [57] | Prospective cohort study | 72 adults in Ethiopia divided in two cohorts: 32 VL/HIV and 46 VL patients | 78.1% of VL/HIV vs 0% in the VL cohort experienced VL relapse within a 3 years period. Immunological features can be utilized as predictors of relapse within the VL/HIV cohort. |
| Fakiola et al, 2013 [58] | Genome wide association study (GWAS) | 1970 individuals: 989 cases from India, 357 cases from Brazil and 1098 controls from India. | The HLA-DRB1–HLA-DQA1 HLA class II region plays a significant role in influencing susceptibility to VL in populations from both India and Brazil. In particular, the association is more pronounced at the single nucleotide polymorphism (SNP) rs9271858 (odds ratio 1.41, 95% C.I. of 1.30-1.52). |
| de Vrij et al, 2021 [59] | Systematic review and preclinical study |  | Variants of the DRB1*15 and DRB1*16 allele groups are associated with lower susceptibility for leishmaniasis. These HLA alleles share common binding motifs within the epitope binding cores, suggesting a potential impact on antigen presentation and immune response. |
| Mahajan et al, 2022 [60] | Cross-sectional study | 1296 people living with HIV infection (PLHIV) > 18 years of age residing in Bahir, India. | Prevalence studies vary based on the diagnostic method. Prevalence of asymptomatic *Leishmania* infection in PLHIV was up to 9%. |
| Guedesid et al, 2022 [61] | Cross-sectional study | 134 participants residing in Brazil of > 18 years old, divided in three groups: 35 symptomatic VL-HIV, 75 asymptomatic VL-HIV and 24 healthy controls. | Asymptomatic individuals are younger, with more years of education and more often on anti-retroviral therapy. Symptomatic individuals presented lower levels of hemoglobin, lymphocytes and CD4 count and higher HIV viral load. Symptomatic patients presented higher serum levels of IL- 17A, IL-6, and IL-10. |
| Giorgio et al, 2020 [62] | Narrative review |  | Granulomatous responses in VL are generally host-protective even though there are some niches within granulomas protecting amastigotes from death. |
| Pampiglione et al. 1974 [63] | Cross-sectional study | 6 asymptomatic infected patients from Northestern Italy identified through serological screening | All 6 patients presented multiple hepatic granulomas on liver biopsy, one of these individuals also presented intracellular *Leishmania* within the hepatic granuloma. |
| Singh et al, 2022 [64] | Cross-sectional study | CD4^+^ T cells of 46 subjects with asymptomatic *Leishmania* infection, 48 VL patients and 59 healthy controls in the region of Bihar, India. | Amphiregulin gene expression, which is a hallmark of T regulatory subsets, was identified as a distinguishing gene product in CD4^+^ T cells from individuals with asymptomatic *L. donovani* infection, compared to VL patients and healthy endemic control individuals. |
| Amprey et al, 2004 [65] | Preclinical study | Mouse models | Mice with deficient NKT cells’ response (CD1d knock-outs) were more susceptible to *L. donovani* infection. NK T cells show a polarized IFN response, implicated in granuloma formation. |
| Venuprasad et al, 2002 [66] | Preclinical study | In vitro models | CD28 expression on neutrophils plays a significant role in both their migration and their ability to produce IFN-γ in response to *L.major* infection. The interaction between neutrophils and macrophages via CD28 and CD80/CD86 contributes to the immune response against the parasite. |
| Alizadeh et al, 2023 [67] | Narrative review |  | Natural killer cells and *Leishmania* interaction. |
| Pitta et al, 2009 [68] | Cross-sectional study | Peripheral blood mononuclear cells taken from 229 participants ranging from 6 to 50 years of age, residing in the eastern part of Sudan. The participants were divided into three groups: asymptomatic subjects, subjects who had been treated for VL and asymptomatic subjects who developed VL during the study period. | L-17 and IL-22 are strongly and independently associated with protection against VL. IL-17 and IL-22 play complementary roles in human protection against VL along with T helper 1 response. A defect in T helper 17 induction increases the risk of VL. |
| Dirkx et al, 2022 [69] | Preclinical study | In vitro and mouse models | Long-term hematopoietic stem cells serve as niches for viable viscerotropic *Leishmania* parasites where they can resist medical treatment. |
| Mandell et al, 2016 [70] | Preclinical study | Mouse models | Mouse models with marked *L.Major* persistent infection can be super-infected by a second marked *L.Major* strain. |
| Mandell et al, 2017 [71] | Preclinical study | In vitro and mouse models | *L.Major* strains persist within mouse tissues while undergoing active replication. |
| Saunders et al, 2020 [72] | Narrative Review |  | The influence of *Leishmania* granulomas on parasite growth is profoundly determined by the host's T helper 1/T helper 2 response and the polarization of both infected and non-infected macrophages towards either a M1 or an M2 phenotype. |
| Terrazas et al, 2017 [73] | Preclinical study | Mouse models | Inflammatory monocytes infected by *L.donovani* display an inflammatory phenotype in the liver and an anti-inflammatory one in the spleen, suggesting chronicization in the spleen. |
| Osorio et al, 2012 [74] | Preclinical study | Hamster models | *L.donovani* directly activates STAT6 mediating an anti-inflammatory environment in the spleen of infected hamsters contributing to impaired control of the infection. |
| Pesseda et al, 2020 [75] | Narrative review |  | Proposed mechanisms explaining the role of arginase in maintaining *Leishmania* infection, including polyamine and thiol synthesis, tissue-resident macrophage (TRM) proliferation and activation and T-cell suppression and exhaustion. |

**Table S2.** Asymptomatic infection of viscerotropic *Leishmania* strains and persistence within the human body - Summary of the articles cited. **Abbreviations**: VL, Visceral Leishmaniasis.

#

| **References** | **Type of study** | **Population** | **Main findings** |
| --- | --- | --- | --- |
| George et al, 2019 [77] | Case report and narrative review | 27-year-old man presented with high-grade intermittent fever for 4 months | The laboratory findings met HLH-2004 criteria and the bone marrow aspirate showed L.*donovani* bodies, suggestive of VL. The man was treated with L-amphotericin and hydrocortisone, but he developed distributive shock and died. |
| Badiola et al, 2020 [78] | Retrospective cohort study | HLH cases in patients over 14 years of age in the province of Granada (Spain), from January 2008 to November 2019. | VL was found to be the causative agent in 20% of all the HLH cases. This percentage is considerably higher than those reported in the previous studies. |
| Jordan, 2023 [79] | Narrative Review |  | HLH is recognized as a maladaptive T cell and innate immune response, mediated by hypersecretion of Interferon (IFN)-γ. In familial forms of HLH (F-HLH), inherited defects of lymphocyte cytotoxic biology underlie excessive T cell activation, demonstrating the importance of the perforin/granzyme pathway as a negative feedback loop. HLH occurring in other contexts and not linked to a genetic predisposition may share some downstream aspects of pathophysiology including excessive IFN-γ levels and activation of innate immune effectors. |
| Tapisiz et al, 2007 [80] | Case report | 2-year-old Turkish boy with VL-HLH | Liposomal amphotericin B was administered and symptoms and clinical findings improved gradually.  *Leishmania* amastigotes are rarely detected on an initial bone marrow smear in cases, as in a recently reported French series of VL-HLH the parasite was not detected in 22% of cases. but it is important to exclude the infection in case of HLH, in order to prevent unnecessary immunosuppressive therapy. |
| Rajagopala et al, 2008 [81] | Case report and systematic review | 23-years-old Indian boy presenting with HLH  Review of 56 VL-HLH reported cases in the English literature | Bone marrow examination revealed hemophagocytosis with no *Leishmania* amastigotes, but anti-rK39 enzyme-linked-immunoassay (ELISA) for leishmaniasis was positive. Amphotericin B desoxycholate was started, with resolution of symptoms. Bone marrow aspiration establishes the diagnosis in 78% of cases but is often negative at onset of the syndrome. |
| Horrillo et al, 2019 [82] | Observational study | 111 Spanish patients with VL, 10% with HIV (HIV-VL), 14% in immunosuppressive treatment (IS-VL), 76% immunocompetent adults (IC-VL); mean age was 45 years. | Fever was experienced by 98% of the IC-VL patients. Plasma ferritin was >1000 ng/ml in 77% of the IC-VL patients vs 17% of the HIV-VL patients. HLH was present in 38% of patients (56% of the IS-VL patients, 18% of the HIV-VL and 37% of the IC-VL patients). Treatment failure was present in the 13.0% patients with VL experienced, eight of whom were in the IC-VL group. |
| Chandra et al, 2013 [83] | Retrospective observational study | 27 cases of VL diagnosed on bone marrow aspirate cytology, India | Pancytopenia (96.2%), anemia (100%), leucopenia (96.2%), thrombocytopenia (96.2%), plasmacytosis (96.2%), hemophagocytosis (70.3%) were the common cytological features while dysmyelopoiesis (3.7%), presence of *Leishmania* bodies in non-histiocytic cells 7.4%), and granuloma with necrosis (10.5%) were uncommon features. |
| Chaturvedi et al, 2021 [84] | Cross-sectional study | American children with either HLH (43, median age 2.6 years old) or sepsis (19, median age 3.0 years old) | Activated (CD38^high^/HLA-DR1) CD8^+^ T cells were found to be expanded in HLH children, not in patients with sepsis. Circulating activated T cells appeared to be broadly characteristic of HLH, with and without genetic lesions or identifiable infections. |
| De Matteis et al, 2022 [85] | Cross-sectional observational study | Blood samples from 74 children followed at Bambino Gesù Children’s Hospital (Italy), with inactive systemic juvenile idiopathic arthritis (sJIA, n=17), active sJIA (n=27), Macrophage activation syndrome (MAS) in sJIA (n=14), infection-associated HLH (n=7) and with other forms of secondary HLH (sHLH) (n=9). | The percentage of CD38^high^/HLA-DR1CD8^+^ T cells in patients with Macrophage activation syndrome (MAS), infection-associated secondary HLH, and other secondary HLH was markedly higher than in patients with sJIA. The presence of a significantly higher percentage of CD8^+^ T cells co-expressing the CD4 marker (CD4^dim^CD8^+^ T cells) in patients with MAS or with other forms of sHLH was another finding. The frequency of CD4^dim^CD8^+^ T cells, but not of CD38^high^/HLA-DR1CD8^+^ T cells, significantly correlated with a clinical severity score, further supporting the involvement of these cells in MAS/sHLH pathogenesis. |
| Nguyen et al, 2024 [86] | Cross-sectional study | Blood samples from patients with hemophagocytic lymphohistiocytosis, hyperinflammation, and immune regulatory disorders | In this study's cohort, the frequency of HLA-DR+CD38^high^ T cells in CD8+ T and CD4+ T effector memory compartments correlated strongly with serum Interleukin-2 receptor levels, as well as ferritin and C-X-C motif chemokine ligand 9 (CXCL9) levels, both at the disease’ onset and during the follow-up. |
| Mastrolia et al, 2023 [87] | Case report | 4 Italian children suffering from HLH secondary to visceral leishmaniasis | Levels of CD38^high^/HLA-DR^+^ cells at the flow cytometry assay were high (36.95% among CD8+ T cells) in all patients at the disease onset and remained high during the disease course (3 out 4 children). A decreasing trend was observed with a complete normalization at 4 weeks (range 3–5) from the HLH onset. |
| Mottaghipisheh et al, 2021 [88] | Retrospective cohort study | 40 children with primary HLH (pHLH) confirmed by genetic study and 20 children with HLH secondary to VL (VL-HLH) confirmed by a blood or bone marrow polymerase chain reaction from Southern Iran. | Patients with pHLH experienced more thrombocytopenia and higher alanine transaminase, while patients with VL-HLH had higher ferritin and erythrocyte sedimentation rate. Central nervous system (CNS) involvement occurred in 38.3% of patients, with no differences between the two groups. The 3-yr overall survival rate was 35.9%. (24% in primary HLH and 100% in VL-associated HLH. |
| Bode et al, 2014 [89] | Retrospective case series study | 13 patients with HLH with imported visceral leishmaniasis, reported to the German HLH reference center | False negative results for *Leishmania* were obtained by initial bone marrow microscopy in 6 out of 13 patients, serology in 1 out of 12 patients, bone marrow culture in 2 out of 5 patients, and polymerase chain reaction (PCR) of peripheral blood in 1 out of 3 patients. Liposomal amphotericin B (L-AmB) was administered to 12 out of 13 patients, Persistent remission was achieved in 11 out of 13 cases, only 2 patients required repeated or prolonged L-AmB therapy. |
| Chellapandian et al, 2013 [90] | Retrospective cohort study | 42 EBV-HLH patients who had received treatment with rituximab-containing regimens associated with other HLH-directed drugs | Rituximab-containing regimens appeared well tolerated and improved clinical status in 43% of patients. Examination of laboratory data obtained prior to and within 2–4 weeks after the first rituximab dose revealed significant reductions in Epstein-Barr Virus load and serum ferritin levels. |
| Cançado et al, 2013 [91] | Case Report | A 72-year-old Brazilian man presenting with HLH | No parasites had been identified in the bone marrow aspirate, but a rK39 rapid dipstick test resulted positive for leishmaniasis. The diagnosis was confirmed by an indirect immunofluorescence test. Liposomal amphotericin B treatment was started and the patient recovered. |
| Matnani et al, 2016 [92] | Case report | 15-year-old boy from Sudan with 2-month duration fever and HLH | Diagnosis of VL was performed with bone marrow aspirate and L-Amphotericin B therapy was started with resolution of fever, splenomegaly, ferritin levels, and recovery of blood counts within 72 hours. |
| Scalzone et al, 2016 [93] | Case report and systematic review | An Italian infant of 7,5 months with HLH | The first bone marrow aspirate was negative for amastigote, as well as the serological test for VL done at the beginning. HLH therapy with corticosteroids and etoposide was started. A bone marrow aspirate was repeated after 3.5 months because of no improvement and *Leishmania* amastigotes were found. L-Amphotericin B was started and overall condition progressively improved. |
| López et al, 2021 [94] | Retrospective cohort study | 127 VL patient from Spain (0–14 years old) | Fever and splenomegaly were the most frequent clinical manifestations (95%). Pancytopenia was observed in 49% of the patients. Anemia was most common (80%), followed by thrombocytopenia (68%) and neutropenia (63%). 29.1% patients developed VL-HLH and they were more common to present pancytopenia. 97.6% of patients were treated with L-amphotericin B, with relapses in 3.1% of cases. There were no differences in terms of total dosage between VL and VL-HLH groups. 35% of VL-HLH patients received immunomodulatory therapy with corticosteroids or cyclosporin and etoposide, in association with antimicrobial therapy and did not have relapses. |
| La Rosée et al, 2019 [95] | Consensus-based expert opinion synthesis |  | HLH triggered by intracellular infections, such as tuberculosis, leishmaniasis, or rickettsial disease, demonstrates favorable response to targeted antimicrobial therapy and immunosuppression as imparted by the HLH-94 protocol should be avoided. |
| George et al, 2018 [96] | Narrative review | Case reports of 25 VL-HLH patients | VL-HLH is a rare condition that mainly affects children (in this review 52% of patients were < 2 years). The common clinical and laboratory features included splenomegaly (100%), hepatomegaly (84%), thrombocytopenia (100%), anemia (96%), leukopenia (84%), hyperferritinemia (68%). Evidence of *Leishmania* infection in the bone marrow was documented in 92% of cases. All patients were treated against *Leishmania*, 80% with L-amphotericin B. 36% of patients were treated also with corticosteroids. |
| Daher et al, 2015 [97] | Retrospective cohort study | 127 Brazilian children with VL, median age 4.2 ± 4.3 years | 27.6% of patients developed HLH during VL progression. The primary symptoms exhibited characteristics consistent with both VL and HLH (fever (100%), splenomegaly (94.2%) and hepatomegaly (60%)). Key laboratory results mirrored the underlying pathophysiology of HLH. Mild cases of acute kidney injury were frequently observed as a typical complication of HLH (45.6%). |
| Blázquez-Gamero et al, 2015 [98] | Multicenter prospective study | 24 Spanish children with VL under 16 years old | 41.7% of patients developed HLH. VL should be excluded in all children with HLH criteria living in or coming from endemic areas. All patients were treated with L-amphotericin B and experienced a positive outcome, regardless of HLH status. Additionally, no discernible distinctions were observed in disease progression between HLH-positive and HLH-negative patients. This indicates that in certain children with VL, secondary HLH may be inherent to the disease's progression, with proper infection management yielding favorable prognosis. |
| Brisse et al, 2016 [99] | Narrative review |  | HLH can be understood as a threshold condition, wherein a combination of genetic and environmental factors leads to escalating inflammation. This process progresses until it reaches a critical point, beyond which immune cell activation and cytokine production become uncontrolled, leading to the characteristic symptoms of HLH. Different pathogenic pathways may converge toward a shared endpoint of severe HLH. |
| Bryceson et al, 2012 [100] | Prospective cohort study | Peripheral blood samples taken from 494 patients referred for evaluation for suspected HLH in 4 European center. Performance of degranulation assays based on surface up-regulation of CD107a on cells and cytotoxic T lymphocytes was evaluated | Patients with familial HLH types 3-5 and certain genetic syndromes (Griscelli syndrome type 2 or Chediak-Higashi syndrome) commonly exhibit abnormal resting Natura killer (NK) cell degranulation, respectively in 97% and 85% of cases. Conversely, patients with X-linked lymphoproliferative disease or Familial hemophagocytic lymphohistiocytosis (FHL) type 2 tend to have normal resting NK-cell degranulation (88%). In cases of secondary HLH, a smaller proportion of patients demonstrate abnormal resting NK-cell degranulation (22%), particularly when compared to those with primary forms of the disease. However, no abnormal degranulation was observed when NK cells were activated by IL-2. |
| Grom, 2004 [101] | Narrative review |  | In familial HLH, the main immune problem is faulty cytotoxic function, notably in NK cells and CD8+ cells. Mutations in the perforin gene are often linked to this issue, reducing perforin expression and impairing the ability of immune cells to kill infected cells. In virus-associated HLH, patients typically exhibit severely reduced or absent NK cell activity, but unlike familial HLH, this is due to significantly decreased numbers of NK cells rather than impaired perforin expression. |
| Al-Samkari et al, 2018 [102] | Narrative review |  | HLH is defined as a dysregulation of cytotoxic T lymphocytes, NK cells and Macrophages. In primary HLH there is an alteration of the cytolytic process linked to genetic alteration of the perforin-granzyme mechanism. Conversely, secondary HLH involves various triggers that perpetuate uncontrolled activation and abnormal function of cytotoxic T lymphocytes and NK cells, creating a proinflammatory environment. |
| Canna et al, 2020 [103] | Narrative review |  | HLH occurs when there is an abnormal activation of phagocytes cells (monocytes, macrophages, and dendritic cells) and type 1 lymphocytes (NK cells, T helper 1, CD8). T cells normally release cytotoxic granules to induce apoptosis of phagocytes and end the immune response. When these granules are impaired or deficient in perforin the activation of mononuclear phagocytes continues unchecked, leading to hemophagocytosis and the release of biomarkers indicative of HLH. |
| Q. Shi et al, 2021 [104] | Retrospective cohort study | 17 VL-HLH patients and 27 VL patients alone admitted at the Beijing Friendship Hospital | VL-HLH patients were more likely to present bleeding, hepatomegaly, low platelet count, elevated triglycerides and ferritin compared to those with VL alone. Moreover, they exhibited heightened inflammation indicated by elevated levels of multiple T helper 1 cytokines (Tumor Necrosis Factor-α, IFN-γ, IL1beta, IL-6, IL-8, IL-12,p70), while in VL patients T helper 2 cytokines (IL-10) prevailed. |
| Machelart et al, 2019 [105] | Case report | A 76-year-old French man with a story of prolonged fever | Based on clinical and laboratory findings, HLH was diagnosticated. First-line infective investigations were negative. Two bone marrow were performed and revealed hemophagocytosis features. Therapy with corticosteroids was initially started but then substituted with etoposide, with improvement. He presented 2 relapses, treated with supplementary doses of etoposide and cyclosporine. Splenectomy was performed and Leishman bodies were found in the histiocytes. VL was diagnosticated and L-Amphotericin B was started, with the recovery of the patient. |
| Gera et al, 2021 [106] | Retrospective cases series | 9 Indian children with HLH, age 6 months - 6 years. | The average age at presentation was 30 months. The most frequent clinical symptoms included fever, hepatosplenomegaly, and pancytopenia. Genetic predisposition was present in 3 children; two had identified infectious triggers (Epstein-Barr Virus and VL). All cases exhibited hyperferritinemia (>1500 ng/mL) and evidence of hemophagocytosis upon bone marrow examination. Children with an inherent genetic predisposition showed significantly heightened ferritin levels (>10000 ng/mL). |
| Mantadakis et al, 2021 [107] | Brief report and mini review | 7-year-old Pomak boy with HLH | Prompt diagnosis of VL was confirmed through bone marrow aspiration. L–amphotericin-B was done with success. Interestingly, the patient had misleading results on tests: a false-negative result for *L.infantum* on enzyme-linked immunosorbent assay and a false-positive result for Epstein Barr Virus (EBV) on immunoglobulin M test. This underscores the importance of ruling out VL, especially in children with a similar age presentation to those with familial HLH, where EBV is a common trigger. |
| Brum et al, 2021 [108] | Case series study | 115 children with VL referred to a Brazilian pediatric infectious disease center | Five cases (4.5%) were confirmed with VL-HLH (median age 2,7 years). All of them presented with fever, splenomegaly, cytopenia, hypertriglyceridemia or hypofibrinogenemia, increased ferritin and hemophagocytosis in the bone marrow. All of them received L-amphotericin B and 4 of them added corticosteroids (2 of them for hemodynamic instability and 1 of them for autoimmune hepatitis) |
| Martín et al, 2009 [109] | Case series study | 3 spanish patients with CGD and VL infection | All of them presented with HLH syndrome at the admission. All of them were treated with L-Amphotericin B: the first one died for multiorgan failure, the other two were treated with IFN-γ therapy in association, with improvement. In some cases, subcutaneous IFN-γ has been proposed as an adjunctive therapy in immunocompromised patients |

**Table S3.** Visceral leishmaniasis-associated hemophagocytic lymphohistiocytosis-mimic - Summary of the articles cited. **Abbreviations**: HLH, hemophagocytic lymphohistiocytosis; IL, Inteleukin; Interferon, IFN, PKDL, Post Kala-azar dermal reaction; VL, Visceral leishmaniasis.

| **References** | **Type of study** | **Population** | **Main findings** |
| --- | --- | --- | --- |
| Dixit et al, 2020 [111] | Narrative review |  | Demonstrating *L*.*donovani* amastigotes in tissue through microscopy is considered the gold standard method for diagnosis, yet it is invasive. Serological evidence, although available, has limited utility due to the persistence of antileishmanial antibodies long after treatment for VL. Polymerase chain reaction assays exhibit high sensitivity (ranging from 76% to 100%) and 100% specificity in diagnosing PKDL when utilizing various targets. |
| Ismail et al, 2006 [112] | Narrative review |  | Ultraviolet B (UVB) light could have a role in pathogenesis of PKDL; in PKDL lesions Langerhans’ cells was decreased and with impaired function (less and shorter dendrites, impaired antigen-presenting ability), as well as after UVB exposure. Also, PKDL lesions exhibit cytokines that are known to be triggered by UVB radiation. |
| Sengupta et al, 2019 [113] | Cross-sectional study | 40 Indian patients with PKDL | In PKDL lesions, there was evident cellular infiltration, including dendritic cells, macrophages (CD68), T-cells (CD8, CD4), and B-cells. Notably, polymorphic lesions showed substantial cellular infiltration, whereas macular lesions displayed milder and patchier infiltration, with the reticular dermis being relatively spared. Furthermore, the parasite load was notably higher in polymorphic lesions compared to macular ones. |
| Singh et al, 2020 [114] | Narrative review |  | Xenodiagnostic assays will play a crucial role in the ongoing efforts to eliminate visceral leishmaniasis (VL) by helping to determine the extent to which human and nonhuman reservoirs contribute to maintaining the transmission cycle of *L.donovani* in endemic regions. |
| Mondal et al, 2019 [115] | Cross-sectional and experimental study designs | 47 PKDL patients and 15 VL patients from Bangladesh, analyzed with xenodiagnosis | 27 PKDL patients (57.4%) tested positive through either direct or indirect xenodiagnosis methods. Patients with positive xenodiagnosis had a higher median skin parasite load than those with negative results. Patients with positive xenodiagnosis had a higher median skin parasite load than those with negative results. |
| Molina et al, 2017 [116] | Observational study | 3 Indian patients with maculopapular or nodular PKDL, analyzed with xenodiagnosis | In all 3 cases, at least 1 sand fly developed detectable infection. Detecting and treating PKDL is a very important aspect of the kala-azar elimination program. |
| Ramesh et al, 2015 [117] | Retrospective observational study | 282 PKDL cases diagnosed between January 1995 to December 2014, at Department of Dermatology, Safdarjung Hospital, New Delhi. Median age 22 years (range 5–65 years). | In 80% of cases there was a delay of more than 12 months between the onset of PKDL and diagnosis. Microscopy exhibited a diagnostic sensitivity of 32-36% both in tissue biopsy and slit-skin smear, whereas Polymerase chain reaction(PCR)/quantitative PCR demonstrated a sensitivity of 96–100%. Treatment adherence surpassed 85% with miltefosine, while it was only 15% with antimonials. The relapse rate with miltefosine was reported to be as high as 13.2%. |
| Gedda et al, 2020 [118] | Narrative review |  | PKDL patients represent a significant reservoir of VL. Up to 20% of VL patients develop PKDL after recovery. Some specific polymorphism of IFNγ receptor reducing its expression have been found in PKDL patients. UVB seem to have a role in the pathogenesis of the lesions, altering the Langerhans cells' morphology and their capacity to present the antigens. |
| Volpedo et al, 2021 [119] | Narrative review |  | In PKDL, the T helper 1 response increases after the VL treatment, with increasing Interferon-γ, Tumor Necrosis Factor-α, and Interleukin-12 levels and decrease in regulatory T cells, Tissue Growth Factor-β and Interleukin-10 levels. Persisting *Leishmania* parasites in the skin trigger reactivated immune cells to infiltrate and cause inflammation in the skin. UVB can contribute to the pro-inflammatory process impacting the function of antigen-presenting cells. This generates suppressor T cells and alters cytokine production. As a result, parasites can replicate in the dermis due to this weakened immune response. |
| Kumar et al, 2014 [120] | Observational study | Venous blood and/or splenic aspirates (SA) samples collected from 84 Indian patients with active VL. All patients were treated with Amphotericin B and cured. Median age 29.45±17.6 | Cytokines in the blood were measured after 6 hours to five days of stimulation with soluble *Leishmania* antigen (SLA). The Interferon-γ response seen was rapid and short lived (the plateau was reached after 18-24 hours and the concentrations were very low after 72 hours). if antigens specific responses could be detected. The same happens in splenic aspirate cultures after stimulation with SLA, indicating that antigen specific cells are present at the site of infection. CD4+ T cells were the source of Interferon-γ in the Western Blot response to SLA in patients with active VL. Following neutralization of Interferon-γ, the parasite load in splenic aspirate increased in 61% of samples. |
| Mukhopadhyay et al, 2015 [121] | Narrative review |  | PKDL patients present a deviation toward T helper 2 (Th2) and Treg response at skin level, exerting a pro-*leishmania* activity. Th2-related cytokines (Interleukin-4, Interleukin-10, and Interleukin-13) promote M2 macrophage polarization, resulting in the release of Tissue Growth Factor-β and Interleukin-10. These molecules inhibit the leishmanicidal activities of M1 macrophages and sustain parasite persistence. Furthermore, M2 macrophages release chemokines that attract T helper 2 and Treg cells, forming a self-reinforcing loop. |
| Mukherjee et al, 2019 [122] | Observational study | 20 Indian patients with PKDL, median age 27.50 years | The histopathological analysis of lesion biopsies showed a dense inflammatory dermal infiltrate, mainly constituted by CD8+ T-cells, a high proportion of CC chemokine ligand 17 (CCL17+) cells (mainly histiocytes) and CC chemokine ligand 22 (CCL22+) cells (dendritic cells). |
| Ismail et al, 2006 [123] | Observational study | 30 Sudanese patients with active PKDL, median age 15 years (range 1–47 years) | The histopathological analysis of lesion biopsies showed that lymphocytes and macrophages infiltrated the dermis, while plasma cells were scarce or absent. CD3+ T cells predominated, with CD4+ cells outnumbering CD8+ cells. Degenerating basal keratinocytes expressed Human Leukocyte Antigen – DR isotype (HLA-DR), Intercellular Adhesion Molecule 1 (ICAM-1), and *Leishmania* antigen, closely interacting with CD4+ T cells. |
| Gasim et al, 1998 [124] | Clinical trial | 29 VL Sudanese patients, successfully treated. | 16 patients developed PKDL during a 2-years follow-up. In this group, a notably high production of Interleukin-10 (IL-10) was observed in keratinocytes and sweat glands, in contrast to the almost complete absence of IL-10 in the skin of patients who did not develop PKDL. The study revealed that PKDL patients exhibited higher levels of IL-10 in their plasma and greater IL-10 production in cultures. In fact, all patients with plasma IL-10 levels exceeding 80 pg/ml ultimately developed PKDL. |
| Ritmeijer et al, 2001 [125] | Randomized Clinical Trial | 199 Ethiopian patients with VL randomly assigned to Pentostam (n = 104) or sodium stibogluconate (SSG) (n = 95) | There were no significant differences between the 2 drugs for the following parameters: frequency of intercurrent events (vomiting, diarrhea, bleeding or pneumonia) or main outcome (death during treatment and death after 6-month follow-up; relapse or PKDL at 6-months follow-up).  The prevalence of PKDL was higher among the 27 individuals who tested positive for HIV compared to those who were HIV-negative, with rates of 27% versus 13% respectively. HIV-positive VL patients had a significantly higher mortality rate (33.3% vs. 3.6%). At the 6-month follow-up, HIV-positive patients exhibited a higher relapse rate (16.7% vs. 1.2%), increased mortality (14.3% vs. 2.4%), and more severe post kala-azar dermal leishmaniasis (27.3% vs. 13.3%) compared to HIV-negative patients. Only 43.5% of HIV-positive patients were considered cured at 6-months follow-up, contrasting with 92.1% of HIV-negative patients. |
| Zijlstra et al, 2014 [126] | Narrative review |  | PKDL is more common and severe in HIV-VL co-infection, not limited to *L.donovani*. Mucosal lesions are common in VL and HIV co-infection, with classical mucocutaneous leishmaniasis being more severe. Immune reconstitution disease is rare in HIV co-infected patients undergoing antiretroviral treatment for leishmaniasis. |
| Salih et al, 2014 [127] | Genome-associated study | Genomic DNA extracted from peripheral blood mononuclear cells of 30 PKDL Sudanese patients | Low expression of Interferon-γ and Interferon-γReceptor 1 (IFNGR1) in PKDL skin biopsies is associated with parasite persistence. In the 841 base pairs of sequence examined, ten variants were discovered in the IFNGR1 initiation site. A cluster of conserved non-coding sequences with putative regulatory variants was identified in the distal promoter of IFNGR1 |
| Dey et al, 2006 [128] | Genome-associated study | Twenty-four strains of *L.donovani* isolated from 18 VL patients and 6 PKDL patients in India | The study identified different polymorphisms in a well-defined genetic locus (b-tubulin) of *L.donovani* DNA. The results showed three different recurrent patterns among 18 VL strains and a genetic homogeneity in all PKDL isolates, suggesting the importance of this specific genetic locus in determining skin or visceral involvement. |

**Table S4.** Immunity and post-kala-azar dermal leishmaniasis - Summary of the articles cited. Abbreviations: PKDL, post-kala-azar dermal leishmaniasis; VL, Visceral Leishmaniasis.
